# Extracellular vesicles derived from dental follicle stem cells regulate tooth eruption by inhibiting osteoclast differentiation

**DOI:** 10.3389/fcell.2024.1503481

**Published:** 2024-12-20

**Authors:** Meng Sun, Yiru Yu, Weixing Zhang, Yi Ding, Ang Li, Ye Li

**Affiliations:** ^1^ Key Laboratory of Shaanxi Province for Craniofacial Precision Medicine Research, College of Stomatology, Xi’an Jiaotong University, Xi’an, China; ^2^ Department of Periodontology, College of Stomatology, Xi’an Jiaotong University, Xi’an, Shaanxi, China

**Keywords:** tooth eruption, dental follicle, extracellular vesicle, osteoclast differentiation, tooth development

## Abstract

Tooth eruption as a crucial part in tooth development and regeneration is accompanied by ongoing osteogenesis and osteoclast activity. The dental follicle (DF) surrounding the developing tooth harbors dental follicle stem cells (DFSCs) which play a crucial role in maintaining bone remodeling. However, the mechanisms through which they regulate the balance between osteogenesis and osteoclast activity during tooth eruption remain poorly understood. Notably, extracellular vesicles (EVs) in bone homeostasis are considered essential. Our study revealed that the DFSCs could modulate tooth eruption by inhibiting osteoclast differentiation via EVs. Further investigation showed that EVs from DFSCs could inhibit osteoclast differentiation through the ANXA1-PPARγ-CEBPα pathway. Animal experiments indicated that EVs from DFSCs and the cargo ANXA1 affected tooth eruption. In summary, this study suggests the critical role of the dental follicle in tooth eruption through EVs, which may provide therapeutic targets for abnormal tooth eruption and effective approaches for the eruption of regenerated teeth.

## Graphical Abstract

**Figure:**
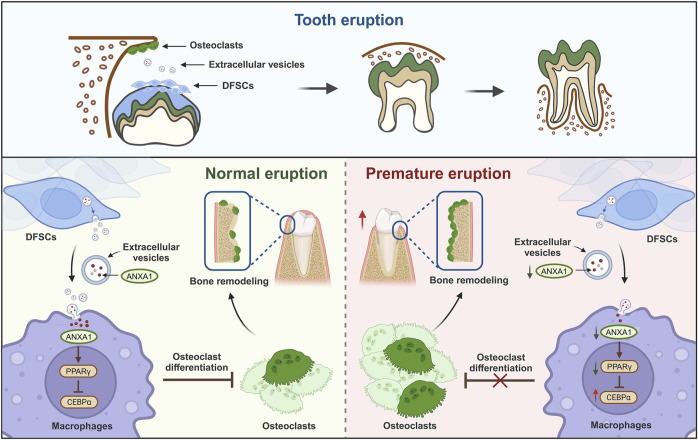
DFSCs regulate tooth eruption by releasing extracellular vesicles. During tooth eruption, dental follicle stem cells inhibit osteoclast differentiation by releasing extracellular vesicles containing ANXA1 and its mediated PPARγ-CEBPα pathway, thereby preventing premature tooth eruption. The elucidation of this mechanism is crucial for understanding and treating abnormal tooth eruption diseases and the eruption process of tooth regeneration. (Image created with BioRender.com, with permission).

## 1 Introduction

Tooth eruption refers to the movement of a tooth from its developmental position through the bone and overlying soft tissues to its functional position in the oral cavity, which is a critical stage in tooth development (Takahashi et al., 2019). Eruption disorders can lead to dental crowding and misalignment, severely impairing the oral function and facial aesthetics of patient (Yamaguchi et al., 2022). Congenital syndromes with abnormal tooth eruption, such as cleidocranial dysplasia and primary failure of eruption, remain poorly understood in terms of pathogenic mechanisms and treatment strategies (Li et al., 2024). Therefore, clarifying the regulatory mechanisms of tooth eruption is of great significance for preventing abnormal tooth eruption and promoting the molecular dentistry of the future.

Tooth eruption entails intricate interactions between the tooth and its surrounding tissues temporally and spatially (Richman, 2019). Among them, the dental follicle tissue, which surrounds the enamel organ and dental papilla, plays an indispensable role in the process of tooth eruption (Chai et al., 2000). Numerous studies have shown that during the intraosseous eruption phase of teeth, the dental follicle exhibits spatiotemporal differences in its regulatory effects on the crown and root (Zeng et al., 2022). Specifically, the root DFSCs can differentiate into osteoblasts under certain conditions, serving as the driving force for tooth eruption, and later forming cementum, periodontal ligament, and the alveolar bone (Morsczeck, 2022). In contrast, the crown dental follicle can recruit monocytes/osteoclast precursors and induce their differentiation into osteoclasts, promoting bone resorption and establishing a pathway for tooth eruption (Bi et al., 2021). However, excessive activity of osteoclasts can lead to abnormal tooth eruption, making it crucial to maintain dynamic balance, the maintenance of which is still not fully understood.

In recent years, extracellular vesicles (EVs) have gained increasing attention as novel mediators involved in bone biological signal transduction, playing a crucial role in bone homeostasis (Liu et al., 2018; Deng et al., 2015). Studies have elucidated the mechanism underlying osteoclast differentiation mediated by EVs among osteoblasts, monocytes-macrophages, and osteoclasts (Huang et al., 2022; Ren et al., 2022). EVs derived from osteoblasts can release RANKL that promote the differentiation of immature osteoclasts into mature osteoclasts (Kobayashi-Sun et al., 2020). However, the role of EVs in tooth eruption remains to be further investigated.

In this study, we demonstrated that EVs derived from dental follicle stem cell (DFSC-EVs) inhibit osteoclast differentiation, thereby regulating the balance of osteoclast during the tooth eruption to ensure normal eruption of tooth. By further elucidating the role of dental follicle tissue in tooth eruption and the regulatory mechanisms, we provide novel theoretical insights into the pathogenesis and treatment of abnormal tooth eruption disorders.

## 2 Methods and materials

### 2.1 Cell isolation and culture

All procedures in the present study were approved by the Ethics Committee of the Institutional Review Board of College of Medicine, Xi’an Jiaotong University (No. KY-GXB-20240001). DFSCs were obtained from dental follicle tissues. Briefly, dental follicle tissues were isolated from an embedded third molar after extraction and incubated in a solution of collagenase type I (Sigma, United States) and Dispase (Roche, CH). Acquired tissues were centrifugated and cultured in α-MEM (Gibco, United States) containing 20% EV-depleted FBS (Procell, CHN) and 1% penicillin-streptomycin (Solarbio, CHN).

RAW264.7 were purchased from Zhongzhouxinqiao Bio-Tech (Shanghai, CHN) and cultured in DMEM (Gibco, United States), supplemented with 10% FBS and 1% penicillin-streptomycin. For osteoclast induction, RAW264.7 were cultured into complete media with 100 ng/mL RANKL and 100 ng/ml M-CSF (PH Biotechnology, CHN).

### 2.2 DFSC-EVs isolation and characterization

DFSC-EVs were enriched as previously described (Li et al., 2023). Briefly, culture supernatants were centrifuged twice at 120,000 × g for 70 min at 4°C and resulting precipitates were -resuspended in PBS. To characterize the isolated DFSC-EVs, TEM was used to observe the morphological identification, NTA was performed to measure diameter distribution and vesicle number of the isolated EVs, and Western blotting was conducted to detect typical EVs markers.

### 2.3 Mass spectrometry

DFSC-EVs were sent to WUWAN BIOBANK (Wuhan, CHN) for protein profiles identification. The further function analysis was performed based on employing Gene Ontology enrichment analysis to detect systematic interpretation of the proteins associated with osteoclast differentiation.

### 2.4 Tartrate-resistant acid phosphatase (TRAP) staining

After Osteoclast differentiation as previously described, RAW 264.7 cells were fixed with 4% paraformaldehyde (Boster, CHN). According to the manufacturer’s instructions, TRAP staining was carried out. Add 800 μL/well TRAP staining solution (Servicebio, CHN) to each well and incubate for 30 min at 37°C. Cells were observed under an inverted light microscopy, and TRAP-positive cells containing three or more nuclei could be counted as osteoclasts.

Mandible bone samples were fixed, decalcified and dehydrated. After paraffin embedding, all specimens were sectioned into 5 μm thick slides. Tissue sections were incubated in TRAP staining solution at 37°C for 20 min. All samples were viewed under a light microscope.

### 2.5 Real-time PCR (RT–qPCR)

The mRNA levels of *ACP5*, *CTSK*, *CFOS*, *ANXA1*, *PPARγ* and *CEBPα* in RAW264.7 was quantified by RT–qPCR. Total RNA of cells was exacted using TRIzol regent (AG, CHN) and then reverse transcription was conducted using SPARKscript Ⅱ RT Plus Kit (SparkJade, CHN) to synthesize cDNA. Finally, the RT–qPCR was performed employing 2×SYBR Green qPCR Mix (SparkJade, CHN). The mean fold changes of gene expression were calculated applying the 2^−ΔΔCt^ method. The sequences of all primers used are provided in Supplementary Table S1.

### 2.6 Western blotting

RAW264.7 were lysed in RIPA buffer (Boster, CHN) to extract proteins and total protein contents were quantified and normalized using bicinchoninic acid Protein Assay Kit (Boster, CHN). Next, the proteins were separated by SDS-polyacrylamide gels (Boster, CHN) and transferred onto a polyvinylidene fluoride (PVDF) membrane (BIO-RAD, United States). After blocked, the membranes were probed with following primary antibodies: rabbit anti-ANXA1 (1:1000; BA0640; Boster, CHN), rabbit anti-PPARγ (1:1000; A00449-3; Boster, CHN), rabbit anti-CEBPα (1:1000; A00386-1; Boster, CHN), rabbit anti-ACP5 (1:1000; A03277-1; Boster, CHN), rabbit anti-CTSK (1:1000; PB9856; Boster, CHN), rabbit anti-CFOS (1:1000; BA0207-2; Boster, CHN), rabbit anti-GAPDH (1:1000; BM3874; Boster, CHN). Then, membrane was incubated with HRP Conjugated AffiniPure Goat Anti-rabbit IgG (1:1000; Boster, CHN) secondary antibodies. Signals were visualized in combination with ECL (NCM Biotech, CHN) and densitometry was performed using ImageJ software.

### 2.7 Animal experiments

Ten-day-old SD rats were divided into different groups: DMSO vehicle control, DFSC-EVs group, GW4869 group, si-ANXA1 group and DFSC-EVs rescue group (each group contains three repeats). DFSC-EVs group and GW4869 group were injected with 2.5 mg/kg GW4869 (MCE, CHN) solution to the first molar tooth dental follicle tissues, and subsequently DFSC-EVs group received an injection of 10 mg/kg DFSC-EVs, or an equivalent volume of 2.5 mg/kg DMSO (Solarbio, CHN) was injected in the DMSO vehicle control. Si-ANXA1 group was treated with si-ANXA1. DFSC-EVs rescue group was treated with si-ANXA1 following injection of 10 mg/kg DFSC-EVs. Ten days after injection, dental follicle tissues were collected and fixed with 4% paraformaldehyde for gross observation, micro-CT assays and histology.

### 2.8 Micro-CT analysis

Whole mandibular first molar and alveolar bones were collected and imaged using micro-CT after fixation in 4% paraformaldehyde for 48 h. According to the size and location of fixed mandible bone samples, 3-dimensional (3-D) measurements and analysis were obtained. The average value of the vertical distance from the highest point of the buccal and tongue to the alveolar ridge was measured as the eruption distance for comparative analysis.

### 2.9 Small interfering RNA transfection

ANXA1 gene expression in DFSC-EVs was silenced by the siRNA transfection. The sense strand of siRNA constructs (Hanbio, CHN) are as follows: hs-ANXA1-si1: 5′-GGU​UAA​AGG​UGU​GGA​UGA​ATT-3′; hs-ANXA1-si2: 5′-GCA​AUU​UGA​UGC​UGA​UGA​ATT-3′; hs-ANXA1-si3: 5′-GCA​GAG​UGU​UUC​AGA​AAU​ATT -3′. siRNA was transfected according to manufacturer’s instructions. Briefly, transfection reagent was incubated with DFSCs at room temperature for 10 min, then the medium was replaced with complete culture medium. Whole cell lysates were used to verify the ANXA1 gene silencing by RT-qPCR at 24–72 h after transfection. DFSC-EVs were isolated to verify the ANXA1 expression by Western blotting.

### 2.10 Immunohistochemistry staining

Mandible bone samples were first fixed in 4% paraformaldehyde and decalcified in 10% EDTA decalcifying solution (Boster, CHN), followed by dehydration with gradient ethanol. After paraffin embedding, all specimens were sectioned into 5 μm thick slides. Tissue sections were incubated with the primary antibodies include anti-Osteopontin/SPP1 antibody (1:100; BM4208; Boster, CHN), anti-PPARγ antibody (1:400; bsm-33436M; Bioss, CHN), anti-CEBPα antibody (1:400; bs-1630R; Bioss, CHN), and the control group was incubated with PBS. Then, the secondary antibodies were individually used to incubate with sections. Finally, staining the slides with DAB (Boster, CHN) and haematoxylin (Boster, CHN). All samples were viewed under a light microscope.

### 2.11 Statistical analysis

Student’s t-test was selected for determine statistical significance between pairwise comparisons, and one-way analysis of variance (ANOVA) was utilized among multiple comparisons. All graphs were plotted with GraphPad Prism software. The statistical significance is indicated by*p < 0.05, **p < 0.01, ***p < 0.001, and ****p < 0.0001.

## 3 Results

### 3.1 DFSC regulated tooth eruption through EVs

**FIGURE 1:**
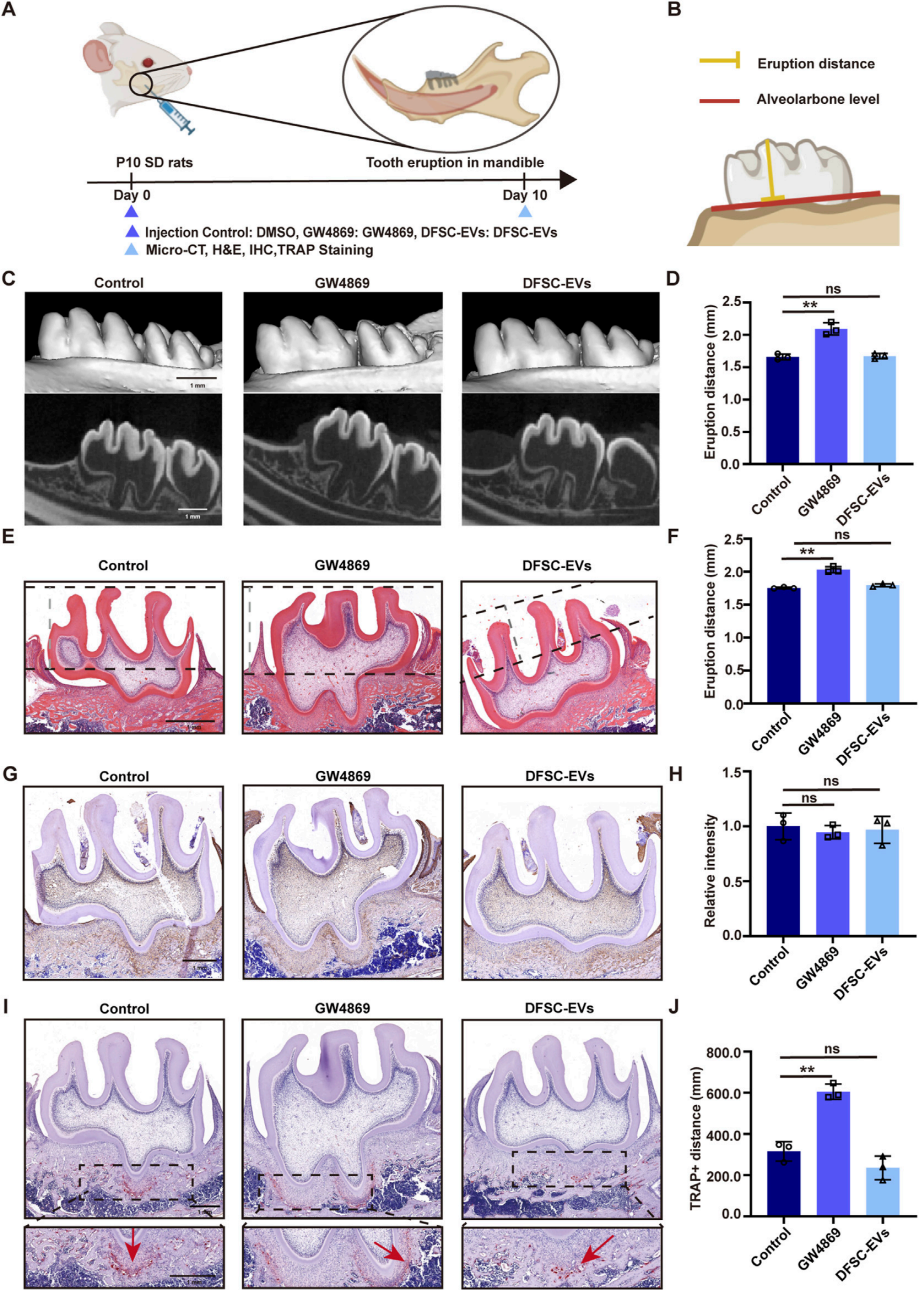
DFSC regulated tooth eruption through EVs. **(A)** Experimental design and schedule for model construction and result assessment. **(B)** Methods of measuring tooth eruption distance. **(C)** Representative micro-CT images of detecting tooth eruption distance. **(D)** Analysis of tooth eruption distance based on micro-CT. **(E)** Representative H&E staining images of the first mandibular molar area. **(F)** Analysis of tooth eruption distance based on H&E staining. **(G)** Representative immunohistochemistry staining images of OPN expression in the first mandibular molar area. **(H)** Quantitative analysis of OPN expression in the first mandibular molar area. **(I)** Representative images of TRAP staining. **(J)** Quantitative analysis of TRAP-positive distance. Scale bar = 1 mm ns, not significant. **p < 0. 01. n = 3.

To explore whether dental follicle tissue play a role in the tooth eruption process by the EVs, we suppressed the secretion of EVs from the dental follicle tissue in SD rats at 10 days post-birth (during the pre-eruptive stage of tooth development) and examined the eruption distance of the first molar (Figures 1A, B). Surprisingly, Micro-CT results showed that compared to the control group, the eruption distance of the teeth significantly increased after adding the exosome secretion inhibitor GW4869, and when DFSC-EVs were reintroduced, there was no significant difference in eruption distance of the first molar compared to the control group, indicating that DFSC-EVs rescued this abnormality (Figures 1C, D). HE staining also showed similar results (Figures 1E, F). To further investigate the cause of abnormal eruption, we conducted immunohistochemical staining for the bone matrix protein osteopontin (OPN) and staining for tartrate-resistant acid phosphatase (TRAP)+ cells (osteoclasts). As shown in Figures 1G, H, there was no significant difference in osteogenic levels among the groups, but after inhibiting DFSC-EVs, the number of osteoclasts in the root area significantly increased. After reintroducing DFSC-EVs, TRAP staining showed no significant difference compared to the control group (Figures 1I, J), suggesting that DFSC-EVs may influence tooth eruption by regulating the differentiation process of osteoclasts.

### 3.2 DFSC-EVs regulated tooth eruption by inhibiting osteoclast differentiation

**FIGURE 2:**
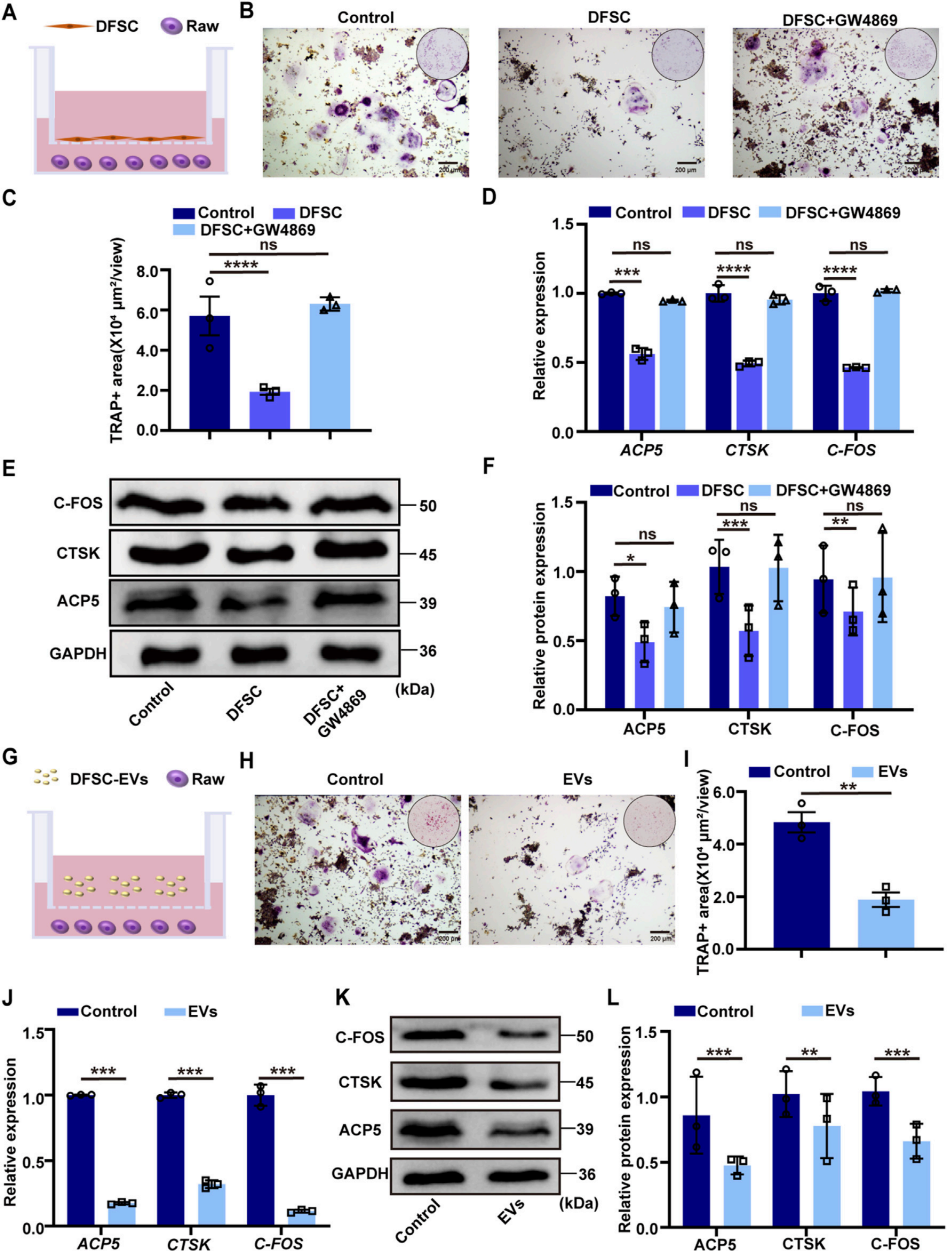
DFSC-EVs regulated tooth eruption by inhibiting osteoclast differentiation. **(A)** Schematic illustration of RAW264.7 and DFSC co-culture system. **(B)** Representative images of TRAP staining. Scale bar = 200 μm. **(C)** Quantitative analysis of TRAP-positive area. **(D)** The mRNA level of *ACP5*, *CTSK* and *CFOS* in RAW264.7 cultured with DFSC. **(E)** The protein level of ACP5, CTSK and CFOS in RAW264.7 cultured with DFSC. **(F)** Western blotting quantification. **(G)** Schematic illustration of RAW264.7 and DFSC-EVs co-culture system. **(H)** Representative images of TRAP staining. Scale bar = 200 μm. **(I)** Quantitative analysis of TRAP-positive area. **(J)** The mRNA level of *ACP5*, *CTSK* and *CFOS* in RAW264.7 cultured with DFSC-EVs. **(K)** The protein level of ACP5, CTSK and CFOS in RAW264.7 cultured with DFSC-EVs. **(L)** Western blotting quantification. ns, not significant. *p < 0.05, **p < 0.01, ***p < 0.001, ****p < 0.0001. n = 3.

To further clarify the mechanism by which DFSC regulates tooth eruption, we examined the effect of DFSC on osteoclast differentiation. The DFSC were identified as expressing mesenchymal stem cell surface markers and exhibiting multi-directional differentiation potential (Supplementary Figure S1). After osteoclast induction, mouse monocyte/macrophage (RAW 264.7) cells were co-cultured with DFSC (Figure 2A), and TRAP staining was used to detect their differentiation into osteoclasts (Figure 2B). As shown in Figure 2C, the area of TRAP-positive regions was significantly decreased after co-culture, indicating a reduction in the number of osteoclasts. While there was no significant difference between the EV-inhibited group and the control group (Figures 2B, C). Further RT–qPCR analysis validated the expression of osteoclast differentiation marker genes *ACP5*, *CTSK*, and *C-FOS* (Figure 2D). Western blotting was employed for further confirmation (Figures 2E, F). The results showed that DFSC could inhibit osteoclast differentiation.

To further investigate whether DFSC regulates osteoclast differentiation through the secretion of EVs, RAW 264.7 cells were co-culture with of DFSC-EVs after osteoclast induction (Figure 2G). TRAP staining results showed a significant reduction in the area of TRAP-positive regions after co-culture with DFSC-EVs (Figures 2H, I). The expression of osteoclast differentiation marker validated by RT–qPCR and Western blotting results showed decreased, which indicated DFSC-EVs inhibited the differentiation of RAW 264.7 into osteoclasts (Figures 2J–L).

### 3.3 ANXA1 was the core factor of DFSC-EVs regulating osteoclast differentiation

**FIGURE 3:**
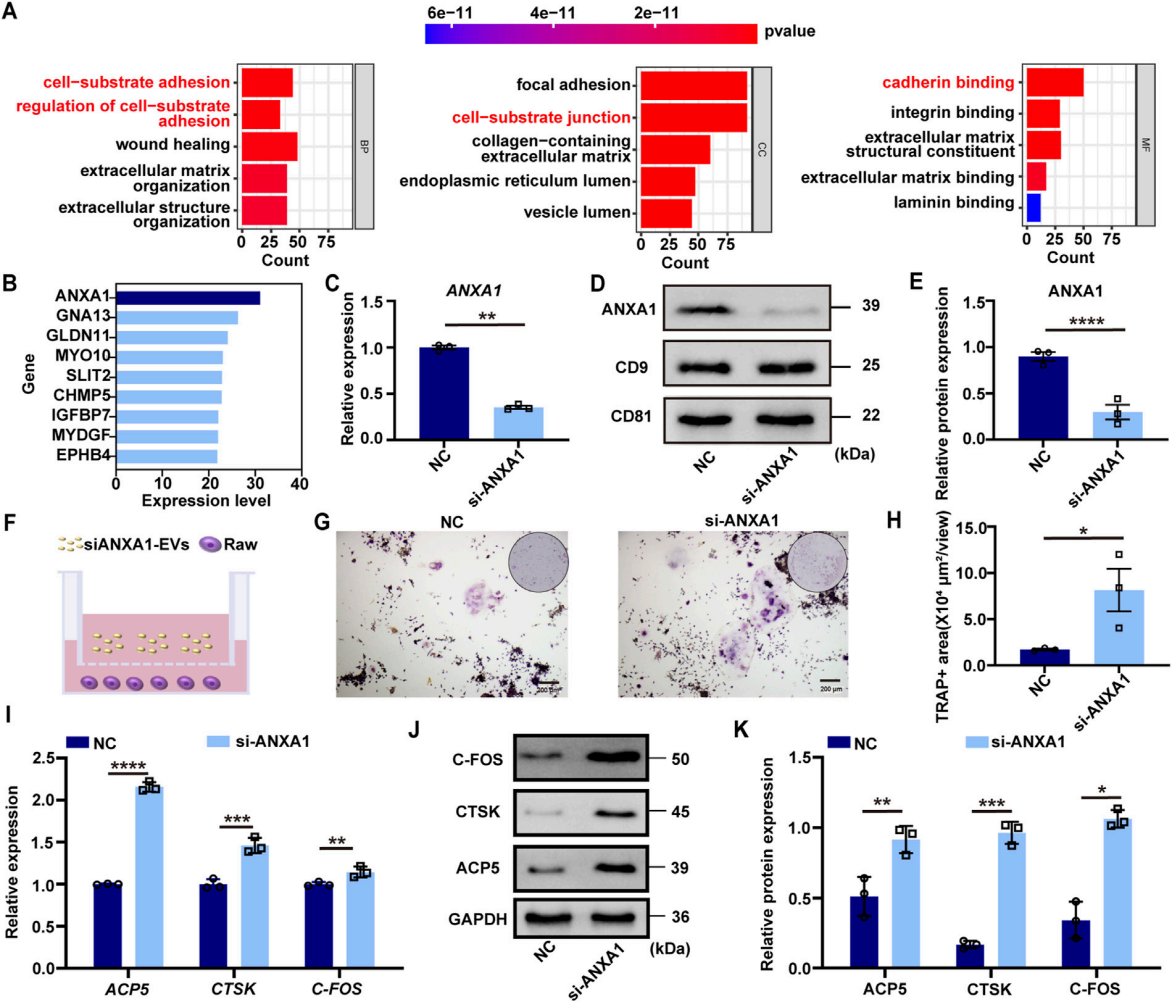
ANXA1 was the core factor of DFSC-EVs regulating osteoclast differentiation. **(A)** Gene ontology enrichment analysis of DFSC-EVs protein profiles. **(B)** The top proteins of Cadherin related to regulating osteoblast differentiation based on expression level. **(C)** The mRNA level of *ANXA1*. **(D)** The protein level of ANXA1. **(E)** Western blotting quantification. **(F)** Schematic illustration of RAW264.7 and siANXA1-EVs co-culture system. **(G)** Representative images of TRAP staining. Scale bar = 200 μm. **(H)** Quantitative analysis of TRAP-positive area. **(I)** The mRNA level of *ACP5*, *CTSK* and *CFOS* in RAW264.7 cultured with siANXA1-EVs. **(J)** The protein level of ACP5, CTSK and CFOS in RAW264.7 cultured with siANXA1-EVs. **(K)** Western blotting quantification. *p < 0.05, **p < 0.01, ***p < 0.001, ****p < 0.0001. n = 3.

To elucidate the molecular mechanism by which DFSC-EVs regulate osteoclast differentiation, we conducted proteomics analysis followed by Gene Ontology (GO) enrichment analysis (Figure 3A). The results indicated that proteins within DFSC-EVs were significantly enriched in biological processes and molecular components related to cell matrix adhesion, in terms of molecular function, these proteins exhibited a notable enrichment in calmodulin-like activities. Among all identified calmodulin proteins related to osteoclast differentiation through mass spectrometry, annexin A1 (ANXA1) was expressed at the highest level (Figure 3B). Small interfering RNA (siRNA) was employed to knock down ANXA1 expression in DFSCs, and Western blotting confirmed successful establishment of low ANXA1-expressing DFSCs (Figures 3C–E). Subsequently, low ANXA1-expressing DFSC-EVs were collected and co-cultured with RAW 264.7 cells after osteoclast induction for TRAP staining (Figure 3F), and the results demonstrated a significant increase in the TRAP-positive area in the si-ANXA1 group (Figures 3G, H). Additionally, RT–qPCR analyses revealed that knocking down ANXA1 resulted in substantial upregulation of osteoclast differentiation marker genes (Figure 3I). Further validation was performed using Western blotting (Figures 3J, K).

### 3.4 ANXA1 mediated PPARγ-CEBPα pathway to regulate osteoclast differentiation

**FIGURE 4:**
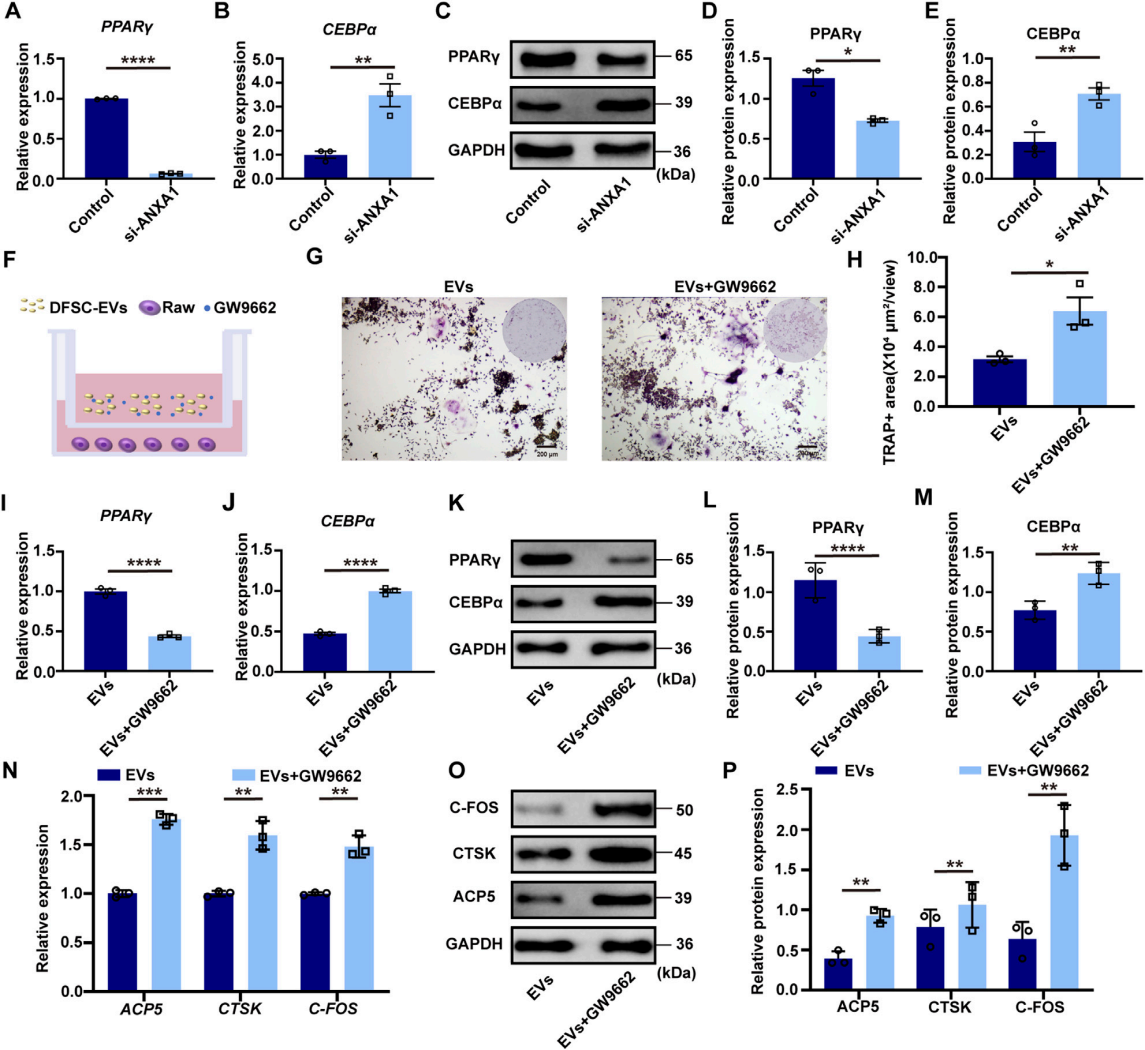
ANXA1 mediated PPARγ-CEBPα pathway to regulate osteoclast differentiation **(A)** The mRNA level of *PPARγ* in RAW264.7 cultured with siANXA1-EVs. **(B)** The mRNA level of *CEBPα* in RAW264.7 cultured with siANXA1-EVs. **(C)** The protein level of PPARγ and CEBPα in RAW264.7 cultured with siANXA1-EVs. **(D)** Quantitative analysis of PPARγ protein expression. **(E)** Quantitative analysis of CEBPα protein expression. **(F)** Schematic illustration of PPARγ inhibited RAW264.7 and DFSC-EVs co-culture system. **(G)** Representative images of TRAP staining. Scale bar = 200 μm. **(H)** Quantitative analysis of TRAP-positive area. **(I)** PPARγ inhibited RAW264.7 construction. **(J)** The mRNA level of *CEBPα* in PPARγ inhibited RAW264.7. **(K)** The protein level of PPARγ and CEBPα in PPARγ inhibited RAW264.7. **(L)** Quantitative analysis of PPARγ protein expression. **(M)** Quantitative analysis of CEBPα protein expression. **(N)** The mRNA level of *ACP5*, *CTSK* and *CFOS* in PPARγ inhibited RAW264.7. **(O)** The protein level of ACP5, CTSK and CFOS in PPARγ inhibited RAW264.7. **(P)** Western blotting quantification. *p < 0.05, **p < 0.01, ***p < 0.001, ****p < 0.0001. n = 3.

It was revealed that ANXA1 inhibits osteoclast differentiation by activating PPARγ expression while concurrently downregulating CEBPα level, which was a key regulator involved in osteoclast lineage differentiation (Perretti and Dalli, 2009; Alhasan et al., 2022; Ma et al., 2023). RT–qPCR and Western blotting confirmed that after knocking down ANXA1, the expression of PPARγ was significantly downregulated, while the expression of PPARγ downstream target CEBPα was upregulated (Figures 4A–E). In the co-culture system of DFSC-EVs and RAW 264.7 cells after osteoclast induction, the PPARγ inhibitor GW9662 was introduced (Figure 4F). TRAP staining illustrated a significant increase in positive areas upon inhibition of PPARγ activity (Figures 4G, H). RT–qPCR and Western blotting results indicated that GW9662 significantly inhibited the expression level of PPARγ in RAW 264.7 after induction while upregulating the expression of its downstream molecule CEBPα (Figures 4I–M). Simultaneously, after inhibiting PPARγ, the mRNA and protein expression levels of osteoclast differentiation marker molecules were significantly upregulated (Figures 4N–P).

### 3.5 DFSCs-EVs/ANXA1 regulating tooth eruption by affecting osteoclast differentiation

**FIGURE 5:**
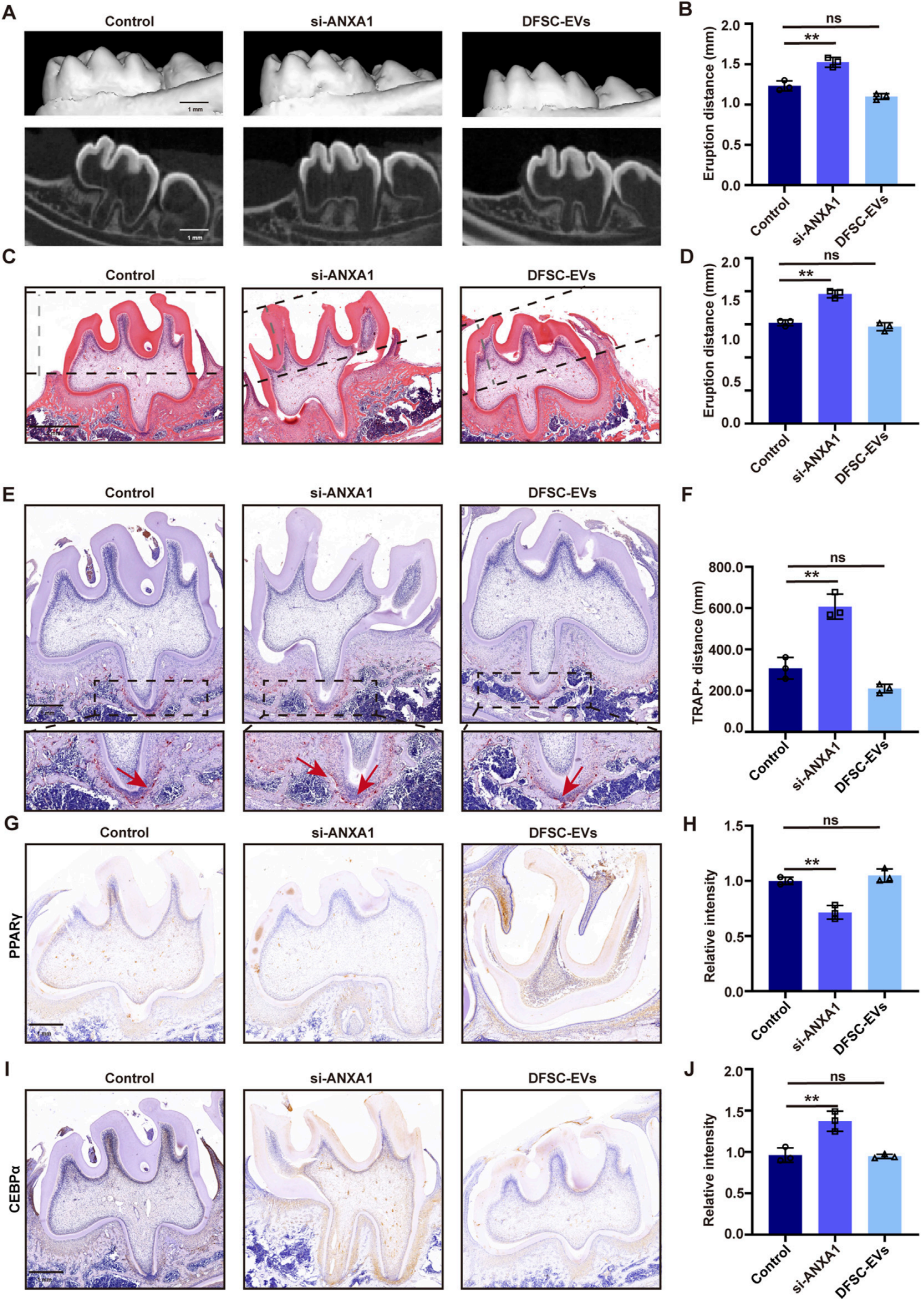
DFSCs-EVs/ANXA1 regulating tooth eruption by affecting osteoclast differentiation. **(A)** Representative micro-CT images of detecting tooth eruption distance. **(B)** Analysis of tooth eruption distance based on micro-CT. **(C)** Representative H&E staining images of the first mandibular molar area. **(D)** Analysis of tooth eruption distance based on H&E staining. **(E)** Representative images of TRAP staining. **(F)** Quantitative analysis of TRAP-positive area. **(G)** Representative immunohistochemistry staining images of PPARγ expression in the first mandibular molar area. **(H)** Quantitative analysis of PPARγ expression in the first mandibular molar area. **(I)** Representative immunohistochemistry staining images of CEBPα expression in the first mandibular molar area. **(J)** Quantitative analysis of CEBPα expression in the first mandibular molar area. ns, not significant. Scale bar = 1 mm **p < 0. 01. n = 3.

*In vivo* animal experiments were conducted to elucidate the role of DFSCs-EVs/ANXA1 in the tooth eruption process. Following si-RNA-mediated knockdown of ANXA1 expression in the dental follicle tissue of SD rats at 10 days post-birth, DFSCs-EVs were injected. Micro-CT analysis revealed a significant increase in tooth eruption distance following ANXA1 knockdown compared to the control group, while reintroducing DFSC-EVs without special treatment resulted in normal eruption distance of the first molar compared to the control group, indicating that DFSC-EVs could rescue the effects of ANXA1 deficiency (Figures 5A, B). HE staining also confirmed these results (Figures 5C, D). Additionally, TRAP staining indicated that knocking down ANXA1 led to an increase in the area of positive regions, while DFSC-EVs effectively restored the level of osteoclast differentiation (Figures 5E, F). Further verification of the expression level of the downstream molecule PPARγ after ANXA1 knockdown by immunohistochemical staining similarly indicated the downregulation of PPARγ, while PPARγ expression was retrieved in the DFSC-EVs group (Figures 5G, H). The expression level of CEBPα, a downstream molecule of PPARγ, was upregulated after ANXA1 knockdown, while DFSC-EVs could downregulate its expression (Figures 5I, J).

## 4 Discussion

In this study, we conducted *in vivo* and *in vitro* experiments to demonstrate that DFSCs in the dental follicle regulate tooth eruption by releasing EVs that inhibit osteoclast differentiation. Mechanistically, DFSC-EVs transported ANXA1 to macrophages, thereby modulating the downstream PPARγ-CEBPα pathway to inhibit their differentiation towards osteoclasts, thus maintaining the homeostasis of bone remodeling during tooth eruption.

The main cells involved in tooth eruption include vascular endothelial cells, epithelial root sheath cells, osteoclasts and dental follicle stem cells (DFSCs). Vascular endothelial cells regulate the permeability of blood vessels and blood circulation to provide nutrients for the cellular metabolism involved in tooth eruption (de Pizzol Júnior et al., 2015). Epithelial root sheath cells are derived from the oral epithelium and surround the dental papilla during root development. They participate in regulating root formation by secreting certain growth factors to induce the differentiation of dental papilla cells and thus participate in the regulation of root formation (Wang et al., 2014). Osteoclasts play a crucial role in the formation of the tooth eruption channel by releasing acidic substances and proteases to dissolve the mineral components and organic matrix of the alveolar bone, creating space for tooth eruption (Wise and King, 2008). While DFSCs play a decisive role in the process of tooth eruption. On the one hand, DFSCs can differentiate into osteoblasts to provide the driving force for tooth eruption. On the other hand, DFSCs can regulate the differentiation of macrophages into osteoclasts to form the tooth eruption channel. The dynamic balance between bone resorption and bone formation during the tooth eruption process is regulated by a complex signal network involving DFSCs (Zhou et al., 2019). Meanwhile, DFSCs can differentiate into cementoblasts, which is also very critical for root development (Zhai et al., 2019). Once DFSCs are removed, tooth eruption is hindered, but the presence of DFSCs still allows replacement inert materials to erupt into the mouth (Marks and Cahill, 1984; Larson et al., 1994). In summary, DFSCs are involved in all the key aspects of tooth eruption and play an indispensable role.

Dental follicle tissue plays a crucial role in regulating asymmetric bone remodeling around the tooth, thereby facilitating tooth eruption (Wise and King, 2008). Previous research has demonstrated that the excision of dental follicle tissue results in the failure of tooth eruption (Marks and Cahill, 1984; Larson et al., 1994; Zhang et al., 2019). While the process of tooth eruption is concomitant with root development and crown mineralization (Wise, 1998). Premature eruption can lead to incomplete mineralization of the crown, subsequently increasing susceptibility to caries (Gozes et al., 2017). Additionally, it may result in tooth looseness or even asphyxiation due to detachment (Posen, 1965). Therefore, maintaining homeostasis in bone remodeling is vital for successful tooth eruption (Gaeta-Araujo et al., 2019). In this study, we identified that DFSC within root dental follicle tissue inhibit premature tooth eruption by suppressing osteoclast differentiation through the release of EVs. This investigation offers new insights into the complex spatio-temporal dynamics exerted by dental follicle tissue on both crown and root during the process of tooth eruption.

Osteoclasts are specialized cells differentiated from monocytes/macrophages playing an important role in maintaining bone homeostasis (Boyle et al., 2003). After adhering to the bone matrix and maturing, osteoclasts secrete acids and lytic enzymes to degrade bone tissue in specific regions (Udagawa et al., 2021). Osteoblasts produce macrophage colony-stimulating factor (M-CSF) to recruit and stimulate osteoclast formation (Takahashi et al., 1988).

In the early stages of tooth eruption, the DFSCs enriched in the dental follicle tissue play a role similar to that of osteoblasts. By day three *postpartum* they can secrete M-CSF as well endothelial-monocyte activating polypeptides, recruiting a large number of monocytes to aggregate in the dental follicle of the mandibular first molar (Wise GE and Fan, 1989). Meanwhile, M-CSF also stimulates the proliferation of monocytes and upregulates RANKL while downregulating OPG expression, significantly increasing the RANKL/OPG ratio, leading to extensive differentiation and activation of monocytes (Marks SC and Cahill, 1987). Subsequently, on day 10 after birth, vascular endothelial growth factor is highly expressed, replacing the role of M-CSF and further promoting osteoclast differentiation (Huang et al., 2016). In this study, we found that DFSCs in the dental follicle could regulate osteoclast differentiation during tooth eruption through the release of EVs, indicating that the regulation of tooth eruption relies on EV-mediated inter-tissue communication.

DFSCs also play a key role in tooth eruption disorders caused by cleidocranial dysplasia, where DFSCs can regulate osteoclast activity through the RUNX2-miR-31-SATB2 pathway, thus affecting the timing and extent of tooth eruption. In patients with cleidocranial dysplasia, a decrease in RUNX2 expression leads to upregulation of miR-31 and downregulation of SATB2 expression, resulting in reduced osteoclast differentiation and activity, ultimately causing delayed tooth eruption (Wang et al., 2016). In our study, the inhibition of EVs secreted by DFSCs and their carried key molecule ANXA1 led to abnormal tooth eruption, while DFSC-EVs could rescue this abnormality, proposing a new mechanism and potential therapeutic targets for abnormal tooth eruption.

## 5 Conclusion

In summary, DFSC-EVs and their carried ANXA1 regulate tooth eruption by inhibiting macrophage differentiation towards osteoclasts through the PPARγ-CEBPα pathway, maintaining the balance of bone homeostasis.

## Data Availability

The original contributions presented in the study are included in the article/Supplementary Material, further inquiries can be directed to the corresponding authors.
