# Supplementary material for: Extracellular vesicles derived from dental follicle stem cells regulate tooth eruption by inhibiting osteoclast differentiation
Source: Front Cell Dev Biol. 2024 Dec 20;12:1503481. doi: 10.3389/fcell.2024.1503481 (PMC11744031; doi:10.3389/fcell.2024.1503481)
Supplement: Supplementary file 1 [file Supplementaryfile1.docx]

Supplementary Material

# Supplementary Figures and Tables

## Supplementary Figures


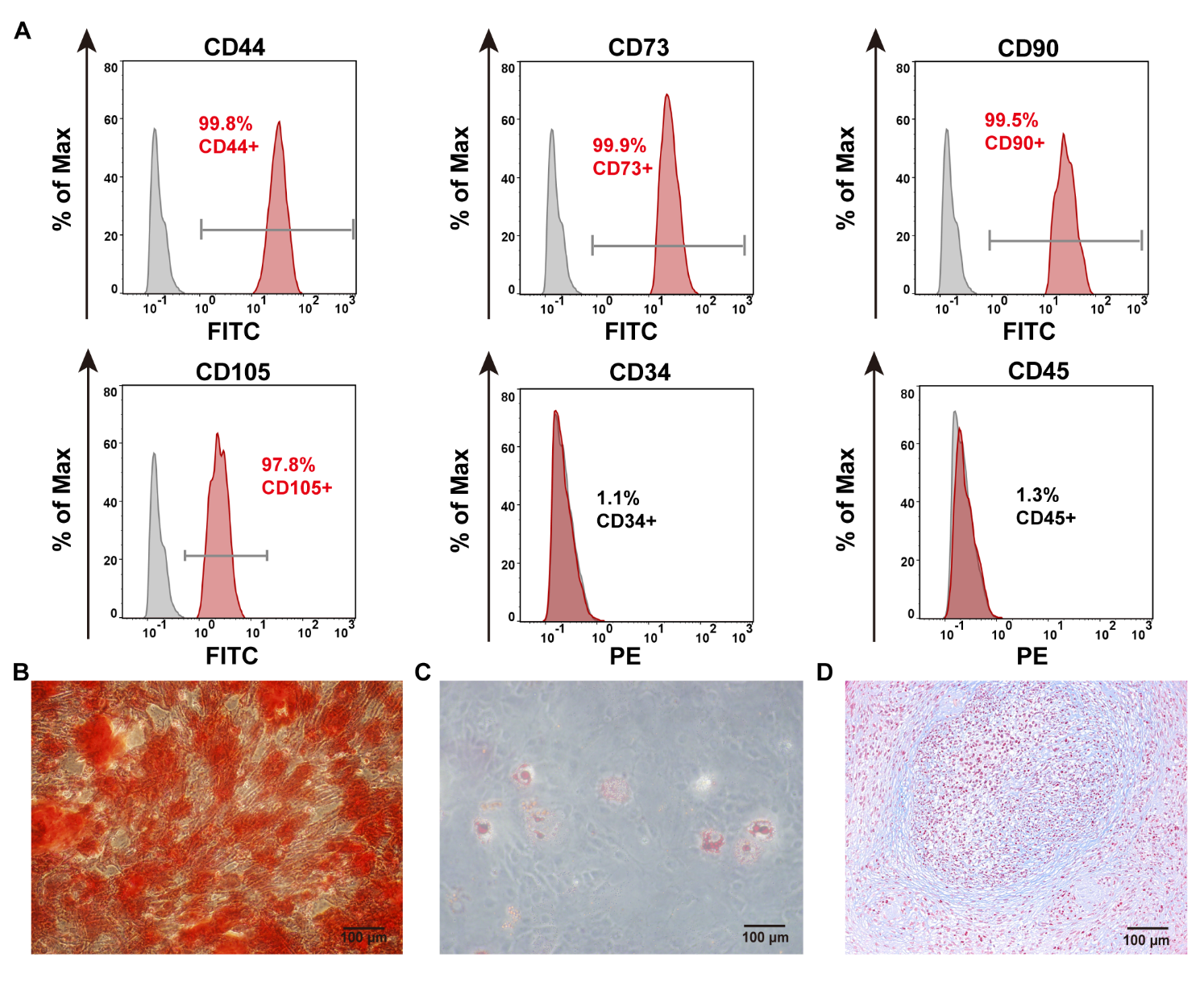


**Supplementary Figure 1.** Characterization of DFSCs.

**(A)** Schematic representation of the expression of DFSC surface markers using flow cytometry. **(B)** Representative images of alizarin red staining after osteogenic induction. **(C)** Representative images of Oil Red O staining showing lipid accumulation after adipogenic induction. **(D)** Representative images of Alcian Blue staining after chondrogenic induction. Scale bar = 100 μm.

**
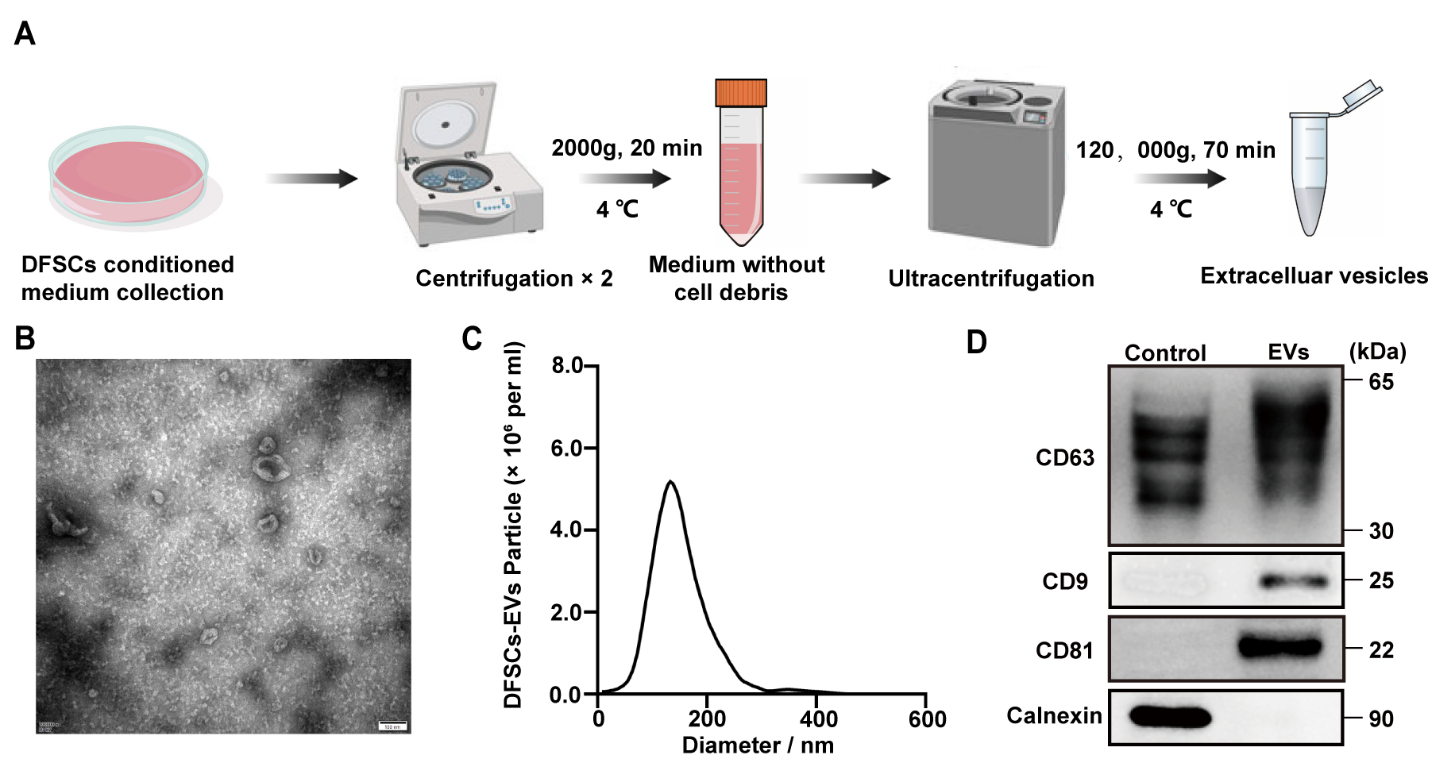
**

**Supplementary Figure 2.** Isolation and characterization of DFSC-EVs.

**(A)** Isolation of DFSC-EVs by Ultracentrifugation. **(B)** Morphology evaluation of DFSC-EVs by TEM. Scale bar = 100 nm. **(C)** Size distribution analysis of DFSC-EVs by NTA. **(D)** Representative marker identification of DFSC-EVs by Western Blotting.

**
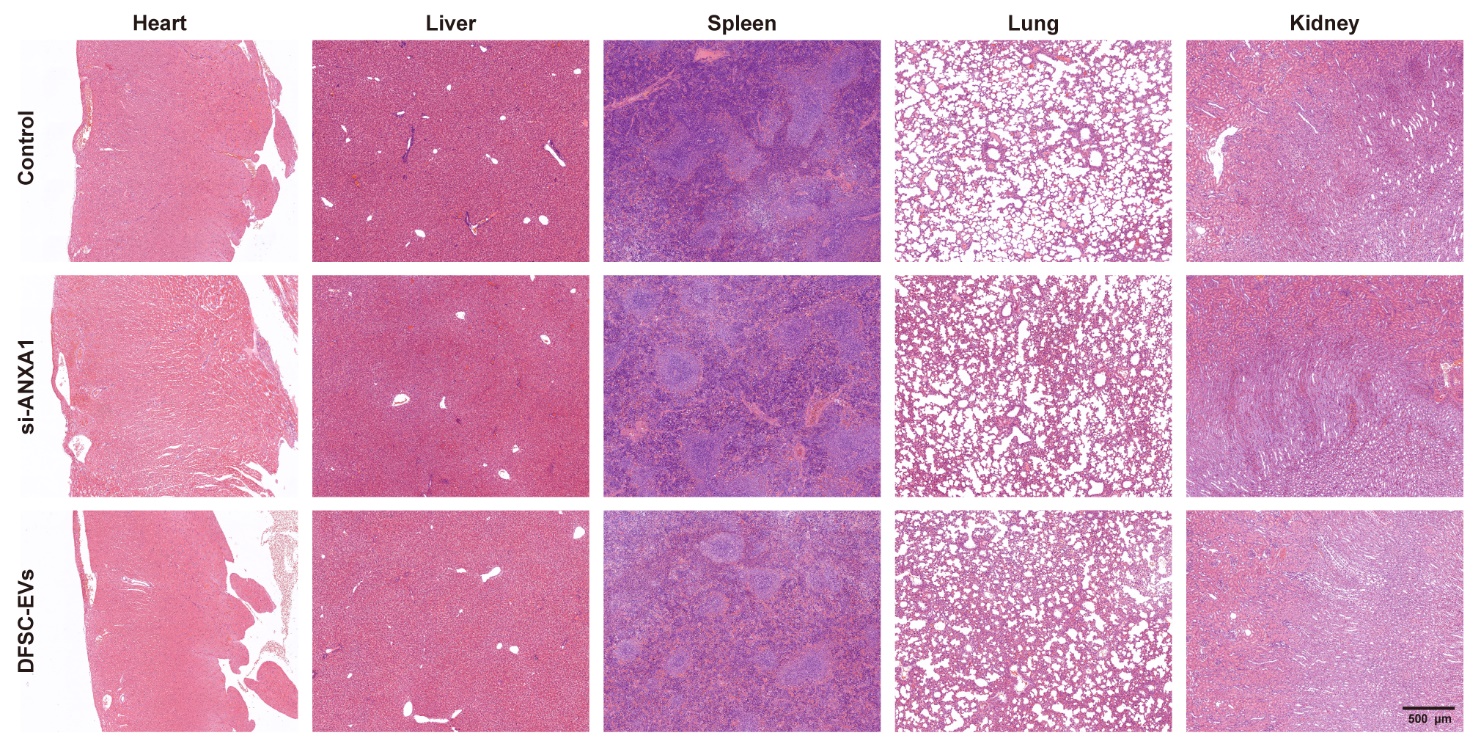
**

**Supplementary Figure 3.** Toxicity test of rat visceral tissues.

## Supplementary Table 1. Sequences of primers used for RT–qPCR in this study.

| Primer names | Sequences |
| --- | --- |
| *ANXA1* forward  *ANXA1* reverse | TGCCGAGAAGCTGTACGAAG  ATTTCCGAACGGGAGACCAT |
| *PPARγ* forward  *PPARγ* reverse | GAGGGCGATCTTGACAGGAA  CACCTCTTTGCTCTGCTCCT |
| *CEBPα* forward  *CEBPα* reverse | AGAAGTCGGTGGACAAGAACA  GGCTTTATCTCGGCTCTTGC |
| *ACP5* forward  *ACP5* reverse | ACCTTGGCAACGTCTCTGCAC  GTCCAGCATAAAGATGGCCACA |
| *CTSK* forward  *CTSK* reverse | AGCAGAACGGAGGCATTGAC  ATTTAGCTGCCTTTGCCGTG |
| *C-FOS* forward  *C-FOS* reverse | TACTACCATTCCCCAGCCGA  GCTGTCACCGTGGGGATAAA |
| *GAPDH* forward  *GAPDH* reverse | GCACCGTCAAGGCTGAGAAC  TGGTGAAGACGCCAGTGGA |

# Supplementary Methods

## Flow cytometry

To characterize DFSC [surface](https://www.sciencedirect.com/topics/immunology-and-microbiology/surface-property) phenotype, cell suspension was incubated with phycoerythrin (PE) or fluorescein isothiocyanate (FITC)- conjugated antibodies (Biolegend, USA). The following conjugated antibodies were used: [CD44](https://www.sciencedirect.com/topics/biochemistry-genetics-and-molecular-biology/cd44), CD73, [CD90](https://www.sciencedirect.com/topics/biochemistry-genetics-and-molecular-biology/cd90), CD105, [CD34](https://www.sciencedirect.com/topics/biochemistry-genetics-and-molecular-biology/cd146) and CD45. Flow cytometry was performed to analyse the percentage of marker-positive DFSCs.

## Osteogenic differentiation and alizarin red staining

The osteogenic medium based on complete medium containing 0.1 µM dexamethasone (MP, USA), 50 µM L-ascorbic acid (MP, USA) and 10 mM β-glycerol phosphate (Sigma, USA). The osteogenic medium was changed twice a week. After 14 days, DFSCs were fixed and incubated with Alizarin Red S solution (Solarbio, CHN). Cells were observed under a microscope to verify the Ca2+ deposits.

## Adipogenic induction and Oil Red O staining

The adipogenic medium based on complete DMEM containing 1 μM dexamethasone (MP, USA), 200 μM indomethacin (Aladdin, CHN), 500 μM 3-isobutyl-1-methylxantine (Beyotime, CHN) and 10 μg/ml insulin (Beyotime, CHN). The adipogenic medium was used for 2–3 days and changed with complete DMEM containing 10 μg/ml insulin for 1 day. Two culture media were consecutively used. Cells were fixed and incubated with Oil Red O stock solution (Solarbio, CHN), then observed under a microscope to visualize lipid droplets.

## Chondrogenic induction and Alcian Blue staining

Chondrogenic differentiation kit (Puhebio, CHN) was used for the chondrogenic induction of DFSCs. Alcian blue staining (Servicebio, CHN) was performed after 21 days. Cell pellets were fixed and embedded in paraffin, then sectioned into 5 μm thick slides. Slides were incubated with alcian blue solution and observed under a microscope.
