# Supplementary figures and images for: Extracellular vesicles derived from dental follicle stem cells regulate tooth eruption by inhibiting osteoclast differentiation
Source: Front Cell Dev Biol. 2024 Dec 20;12:1503481. doi: 10.3389/fcell.2024.1503481 (PMC11744031; doi:10.3389/fcell.2024.1503481)

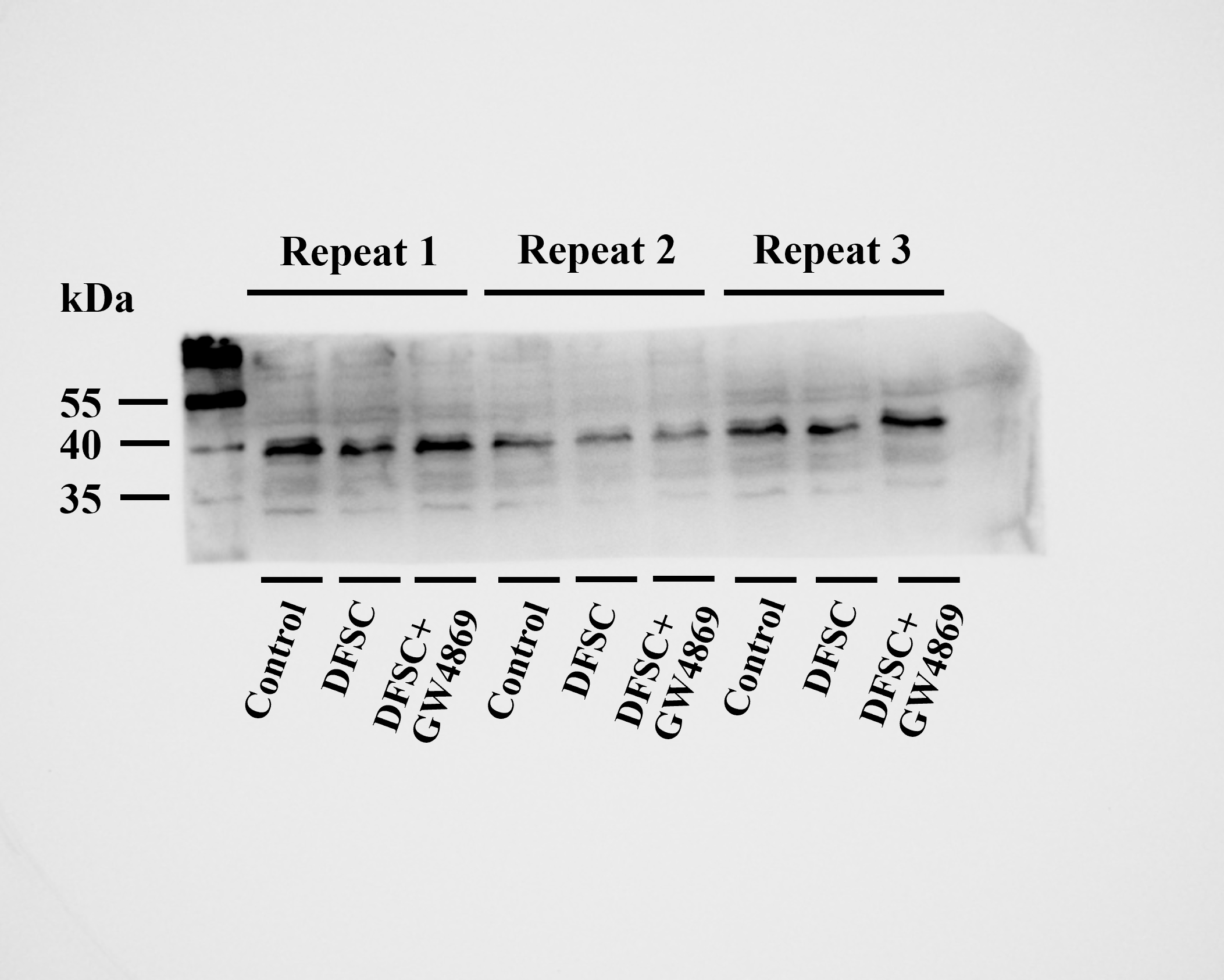

Supplement: Supplementary file 2 [file DataSheet1.zip › Western Blot_raw_images/Figure 2/E/ACP5.tif]

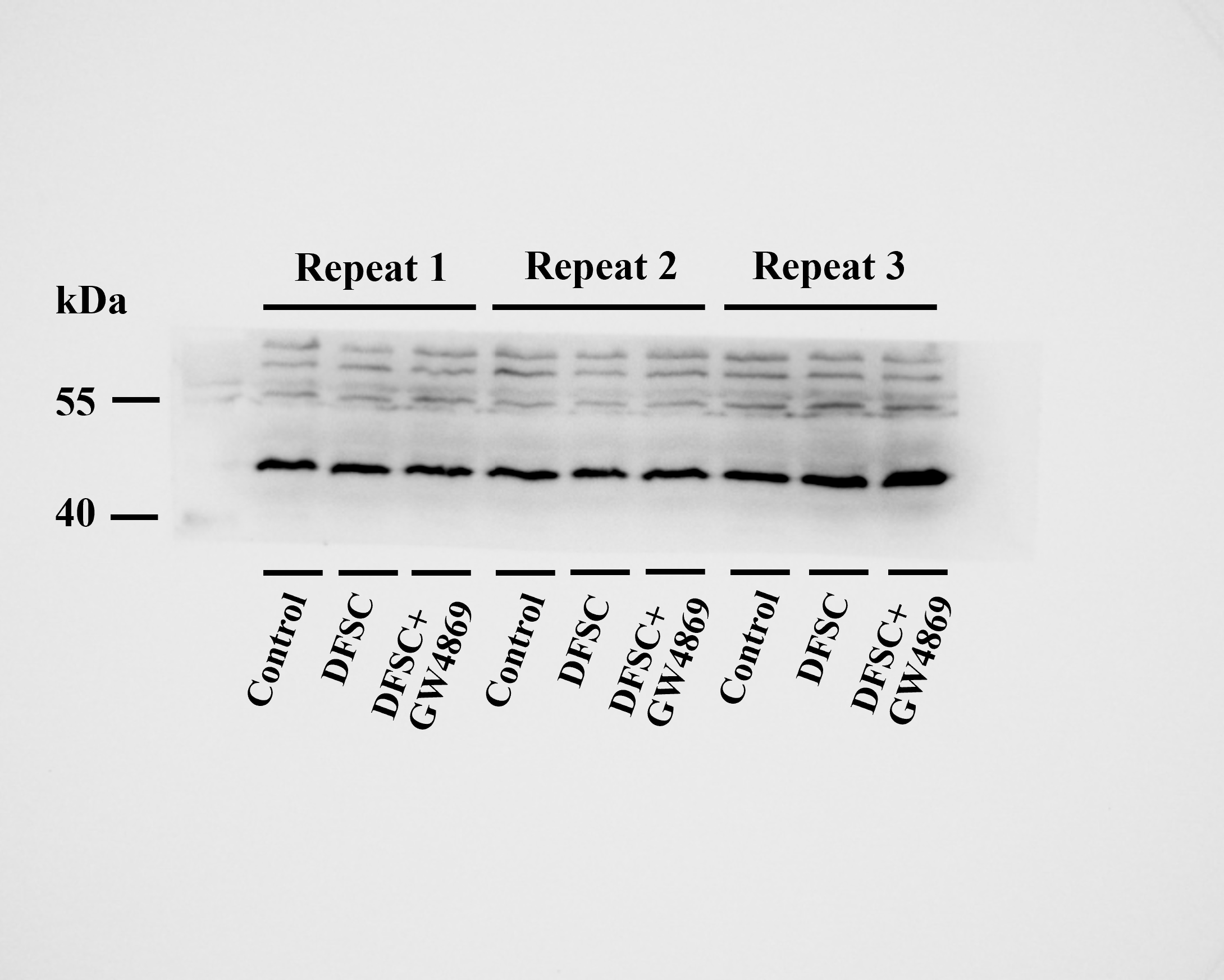

Supplement: Supplementary file 2 [file DataSheet1.zip › Western Blot_raw_images/Figure 2/E/CFOS.tif]

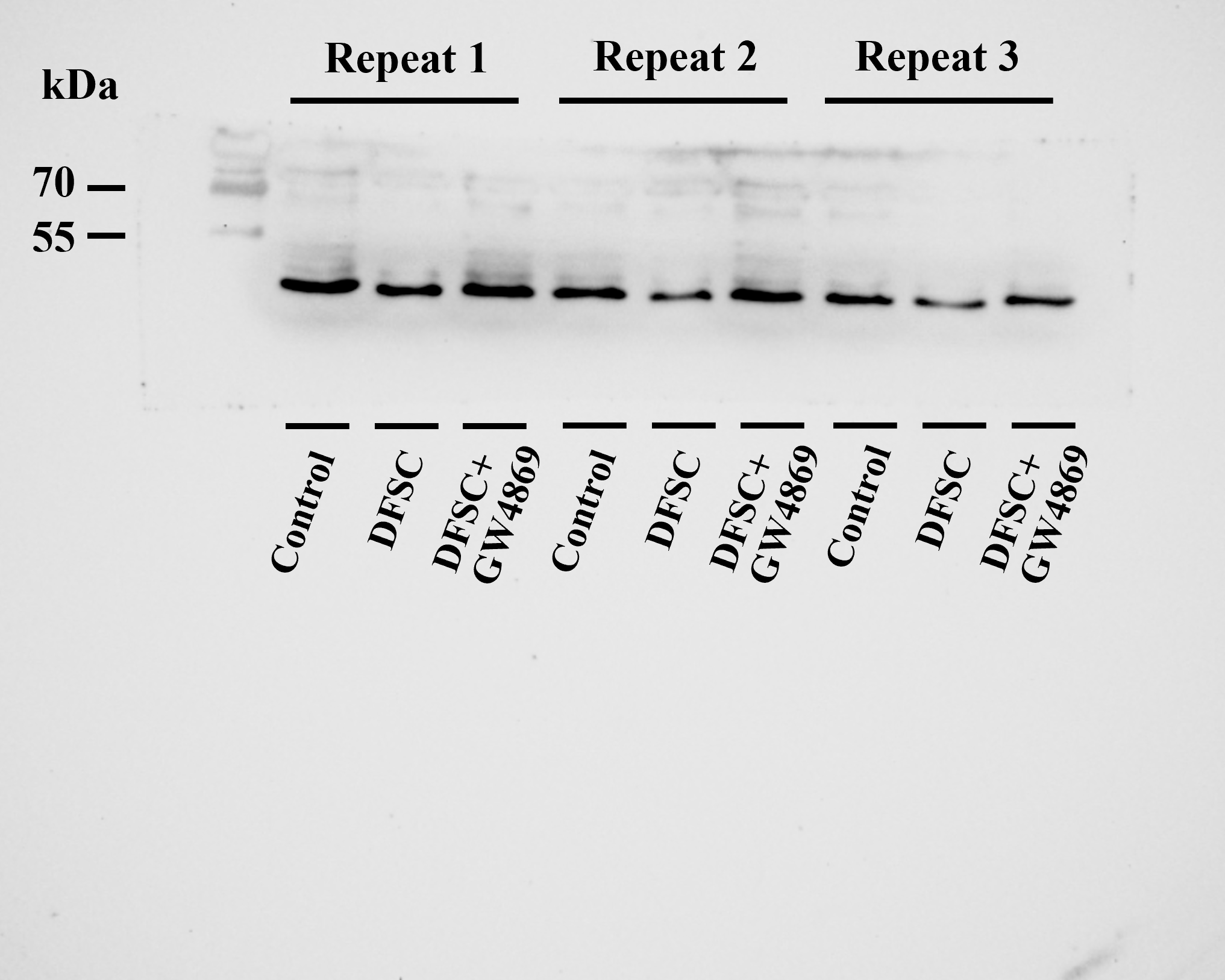

Supplement: Supplementary file 2 [file DataSheet1.zip › Western Blot_raw_images/Figure 2/E/CTSK.tif]

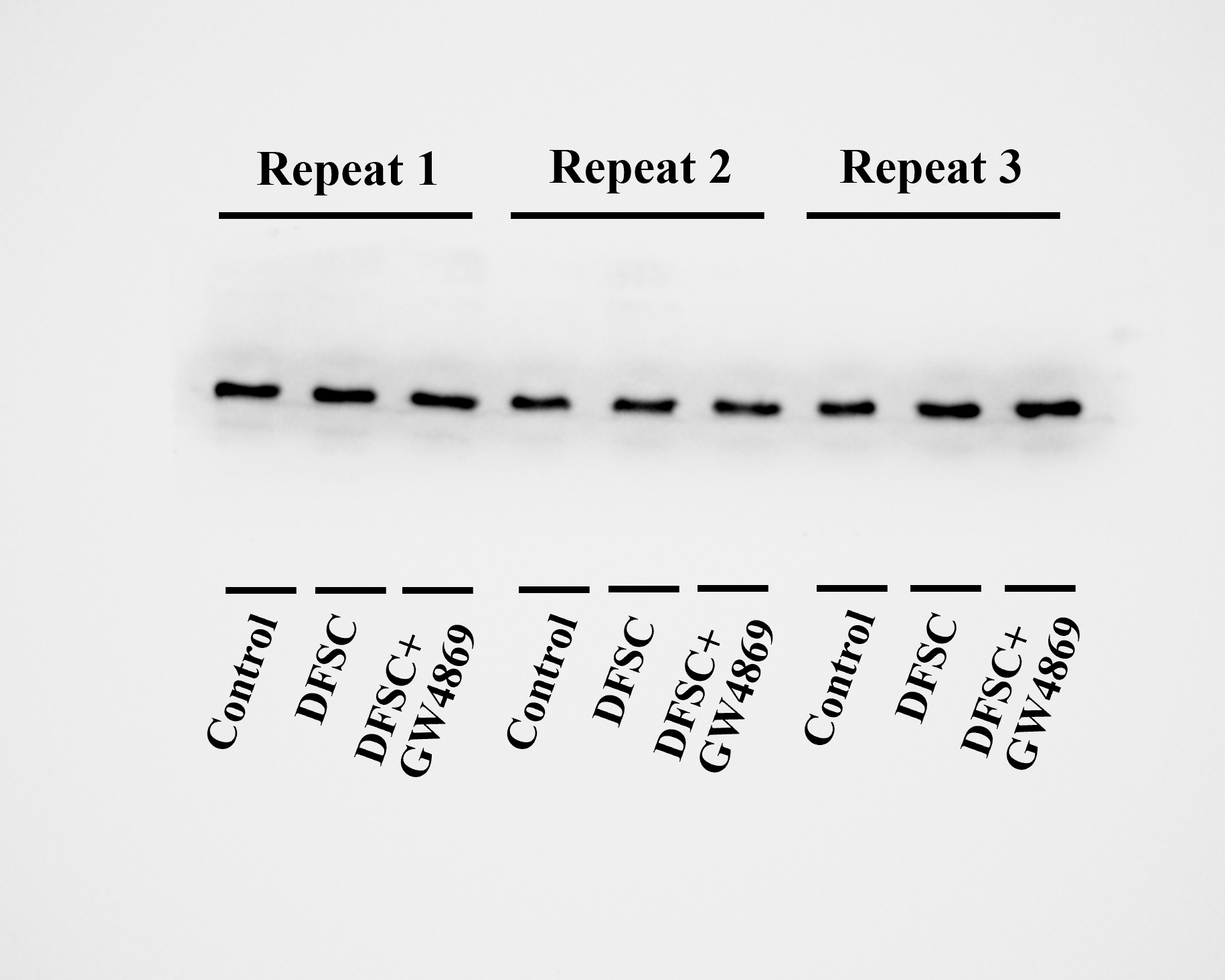

Supplement: Supplementary file 2 [file DataSheet1.zip › Western Blot_raw_images/Figure 2/E/GAPDH.tif]

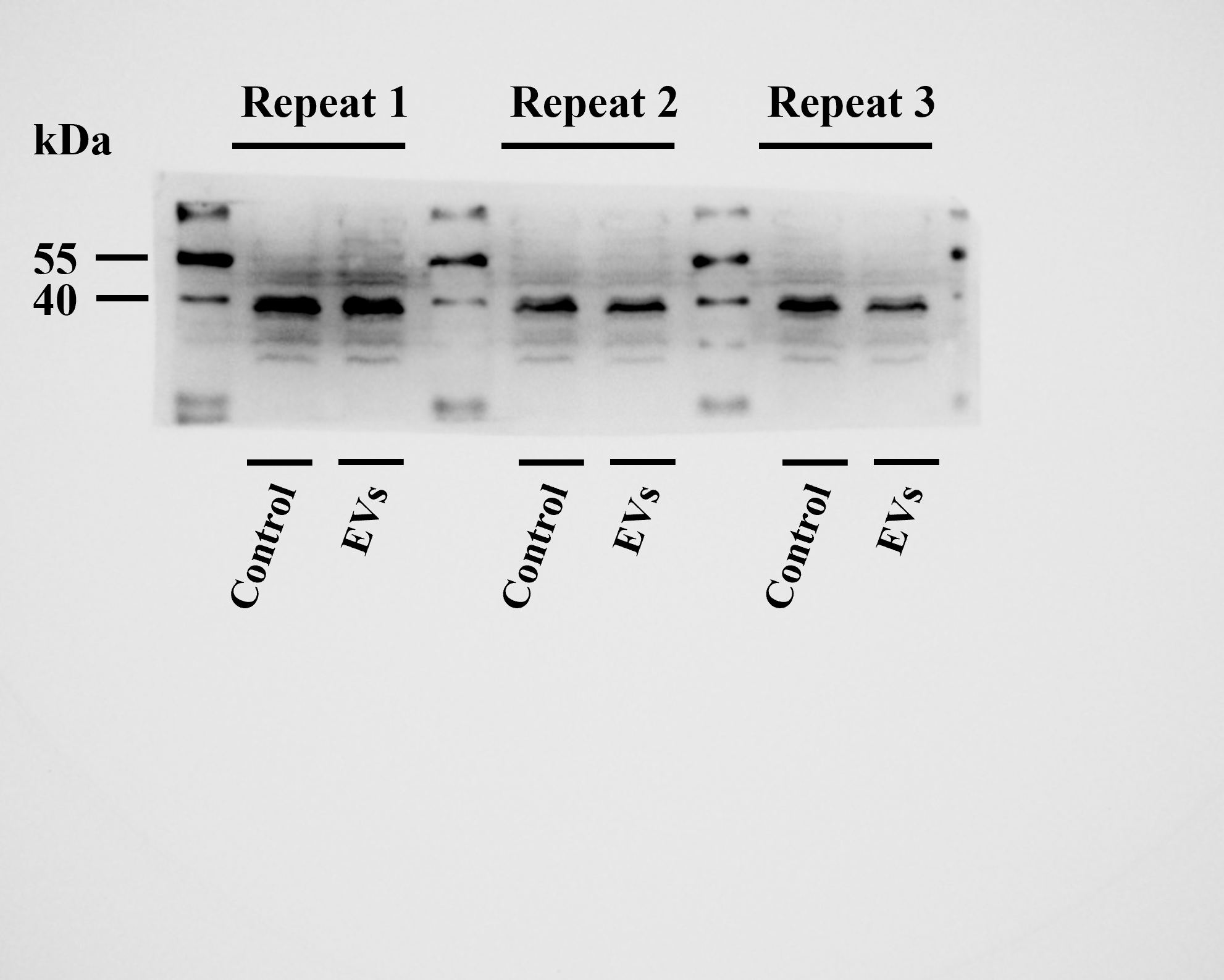

Supplement: Supplementary file 2 [file DataSheet1.zip › Western Blot_raw_images/Figure 2/K/ACP5.tif]

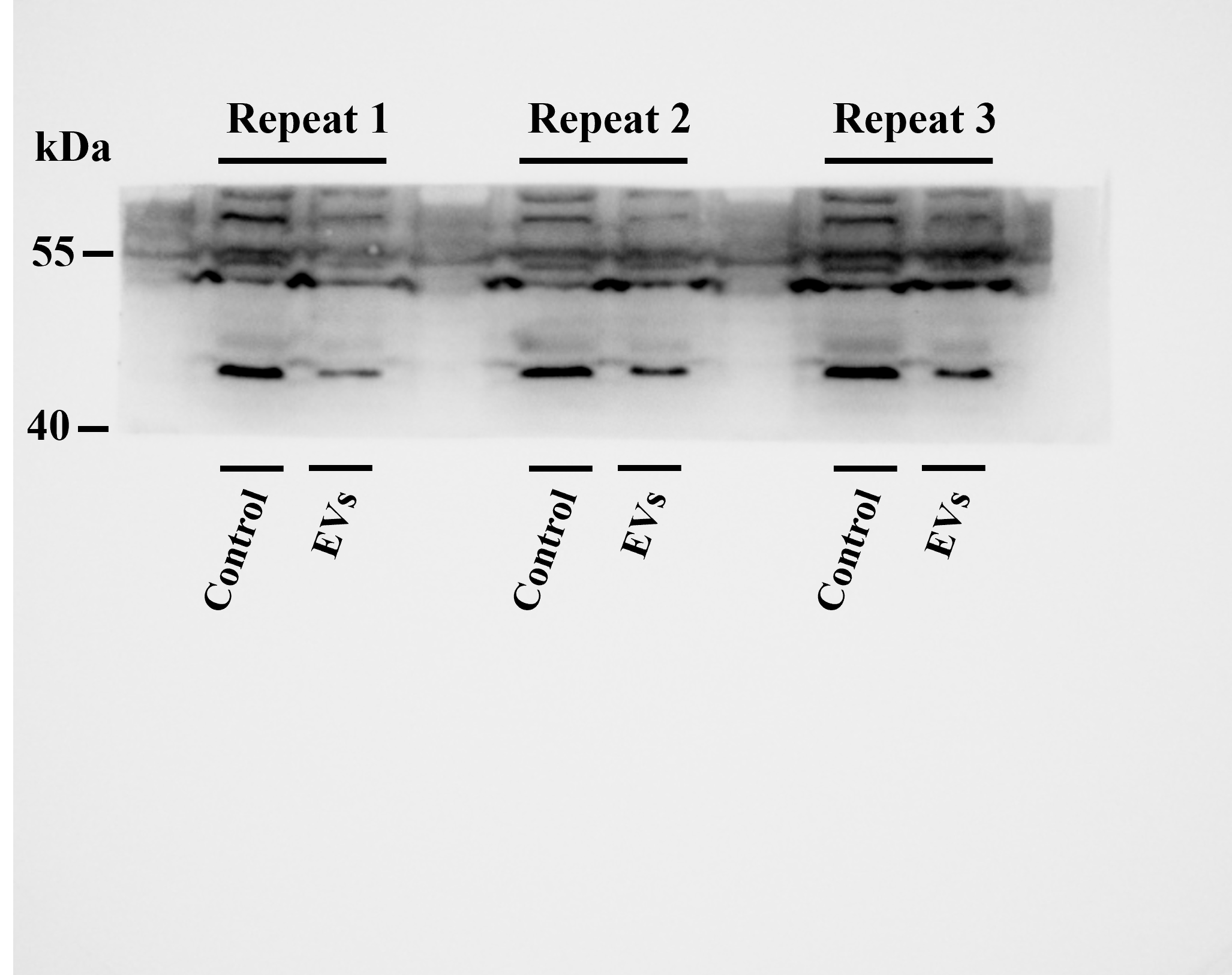

Supplement: Supplementary file 2 [file DataSheet1.zip › Western Blot_raw_images/Figure 2/K/CFOS.tif]

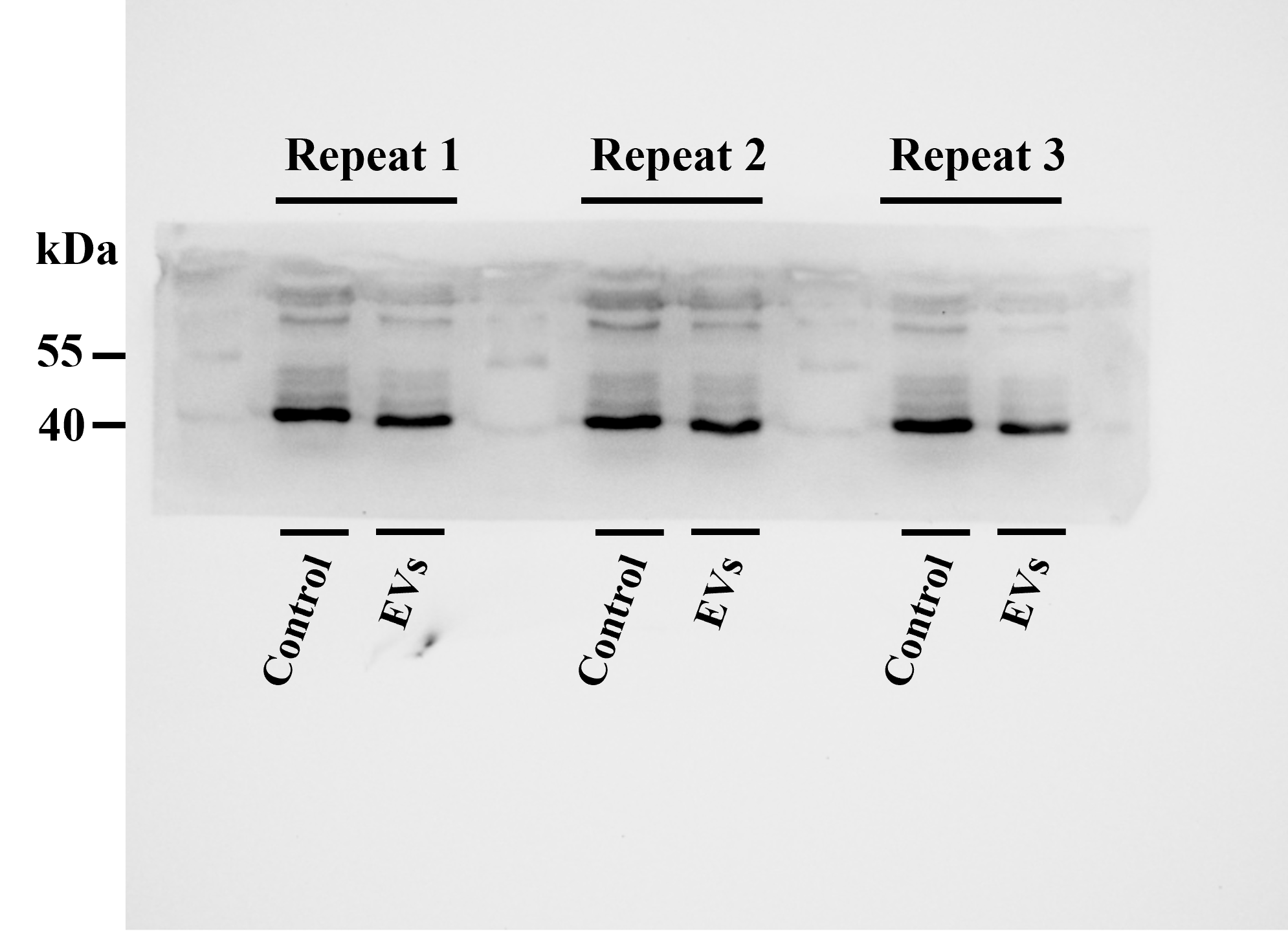

Supplement: Supplementary file 2 [file DataSheet1.zip › Western Blot_raw_images/Figure 2/K/CTSK.tif]

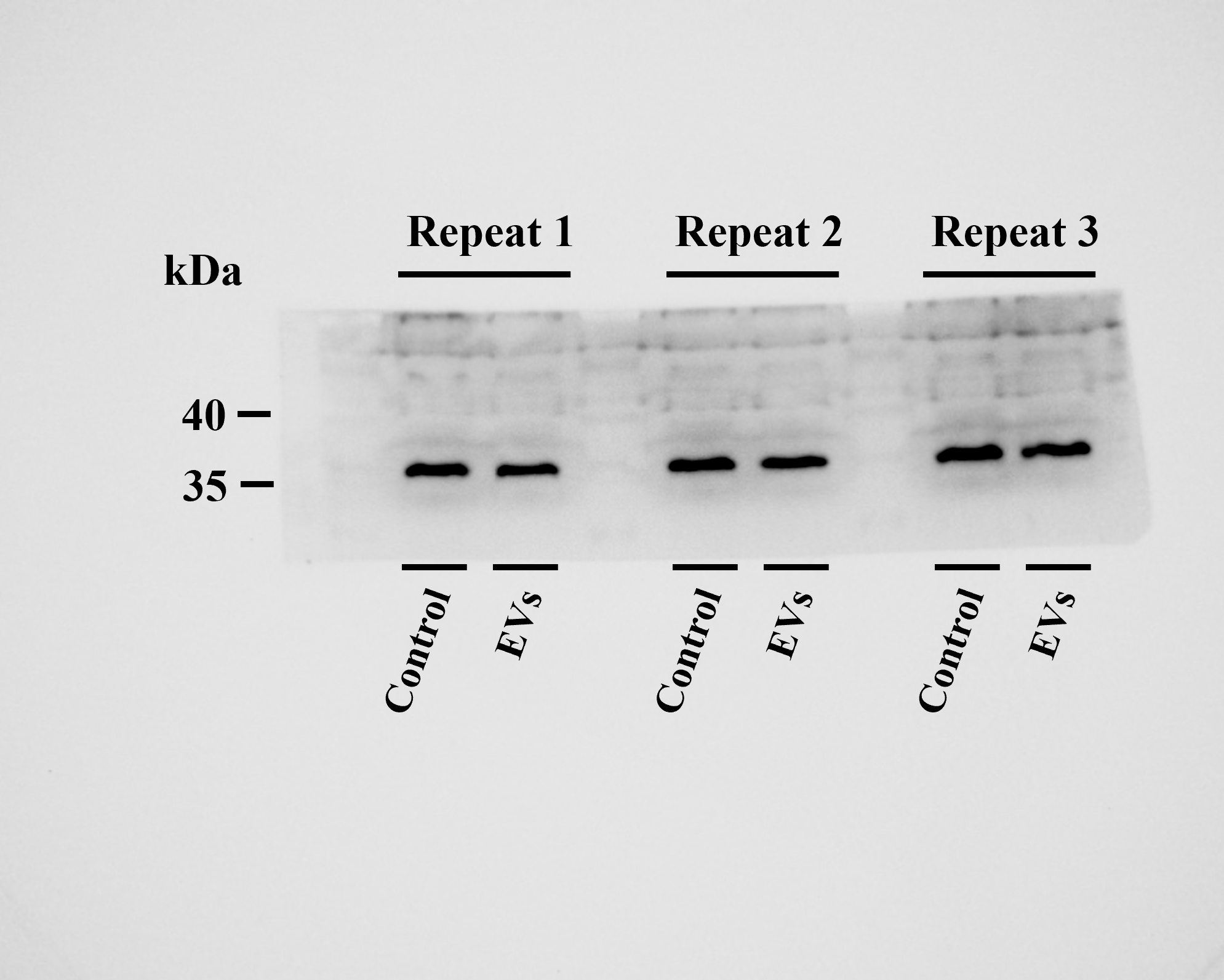

Supplement: Supplementary file 2 [file DataSheet1.zip › Western Blot_raw_images/Figure 2/K/GAPDH.tif]

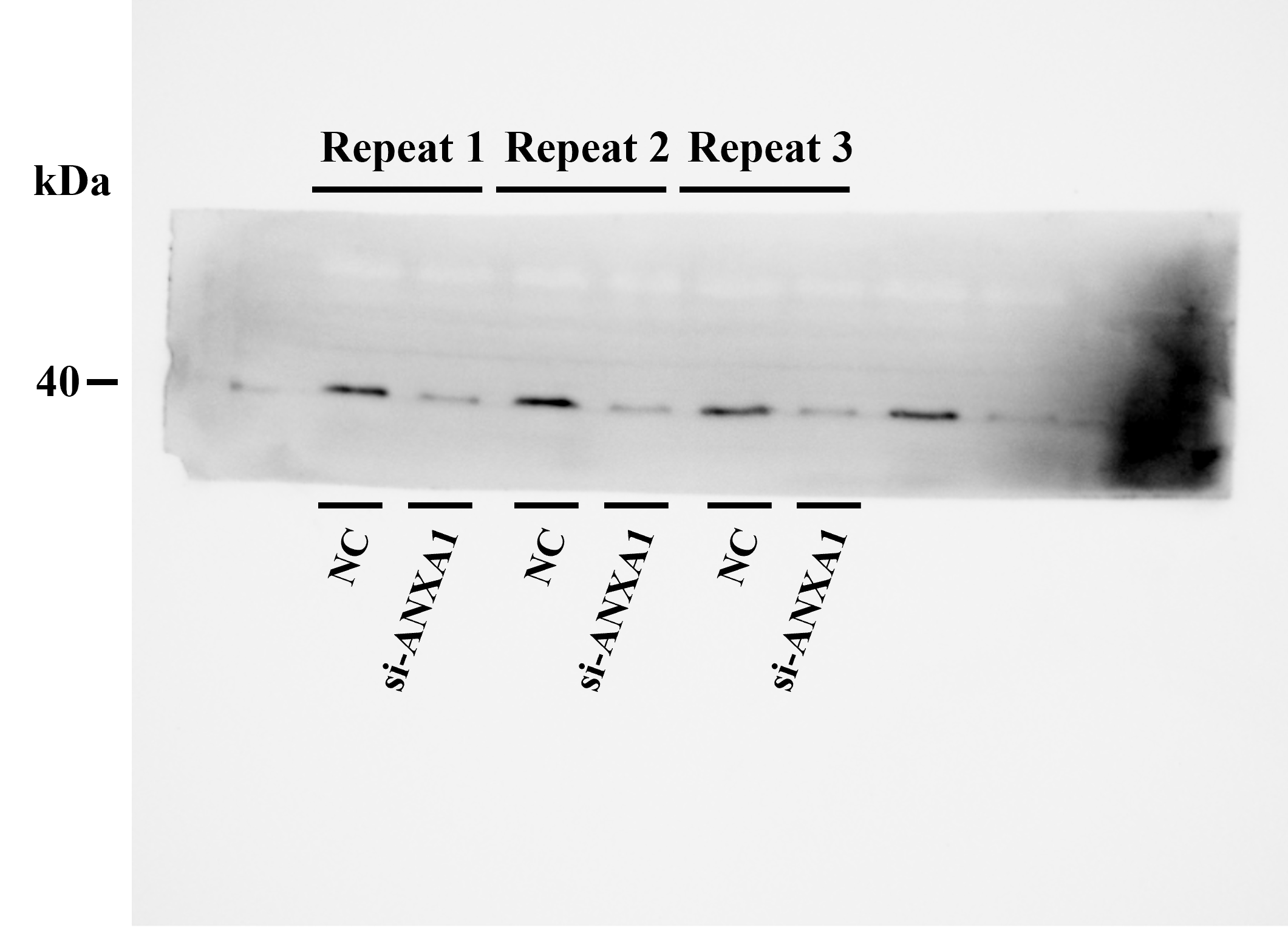

Supplement: Supplementary file 2 [file DataSheet1.zip › Western Blot_raw_images/Figure 3/D/ANXA1.tif]

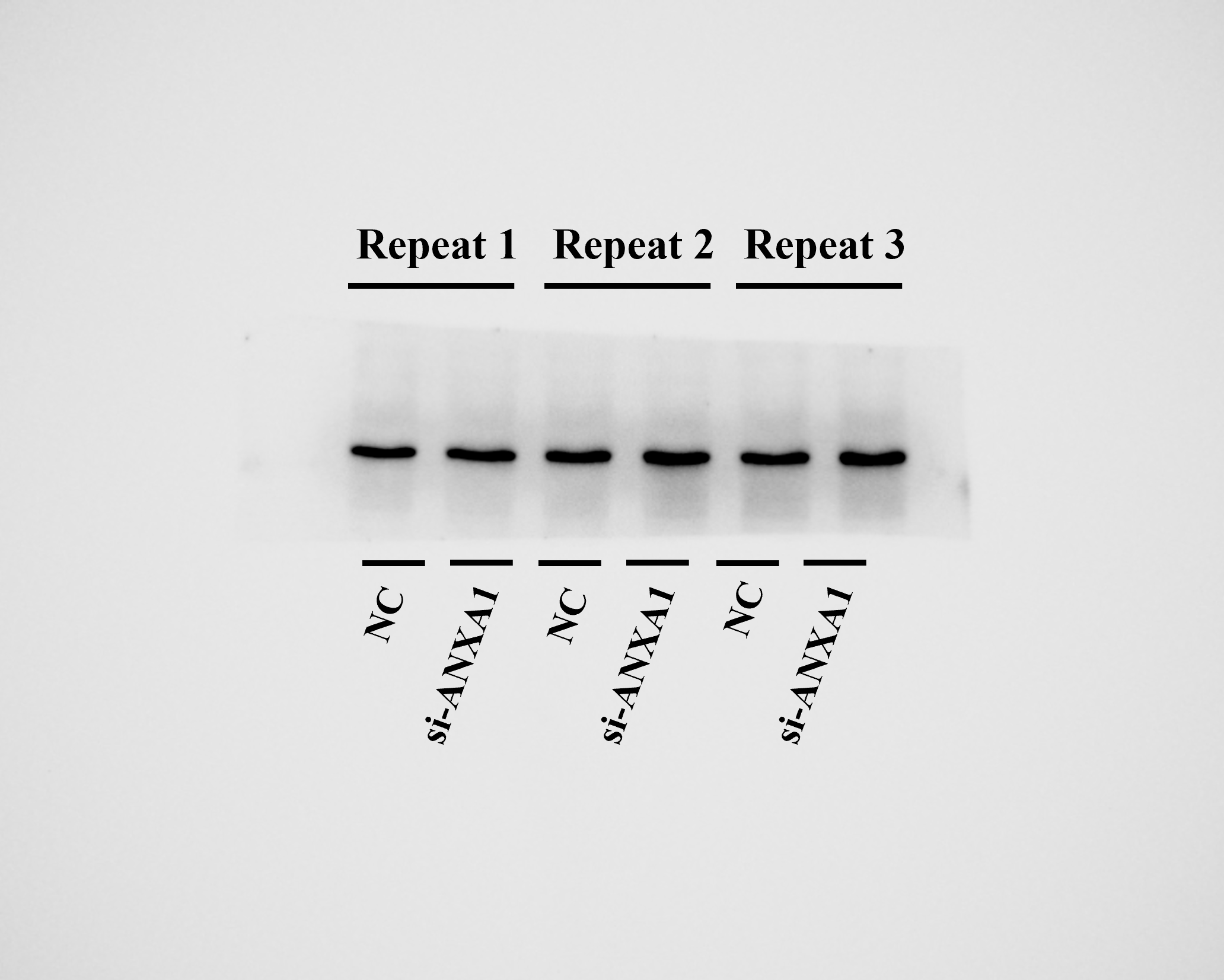

Supplement: Supplementary file 2 [file DataSheet1.zip › Western Blot_raw_images/Figure 3/D/CD81.tif]

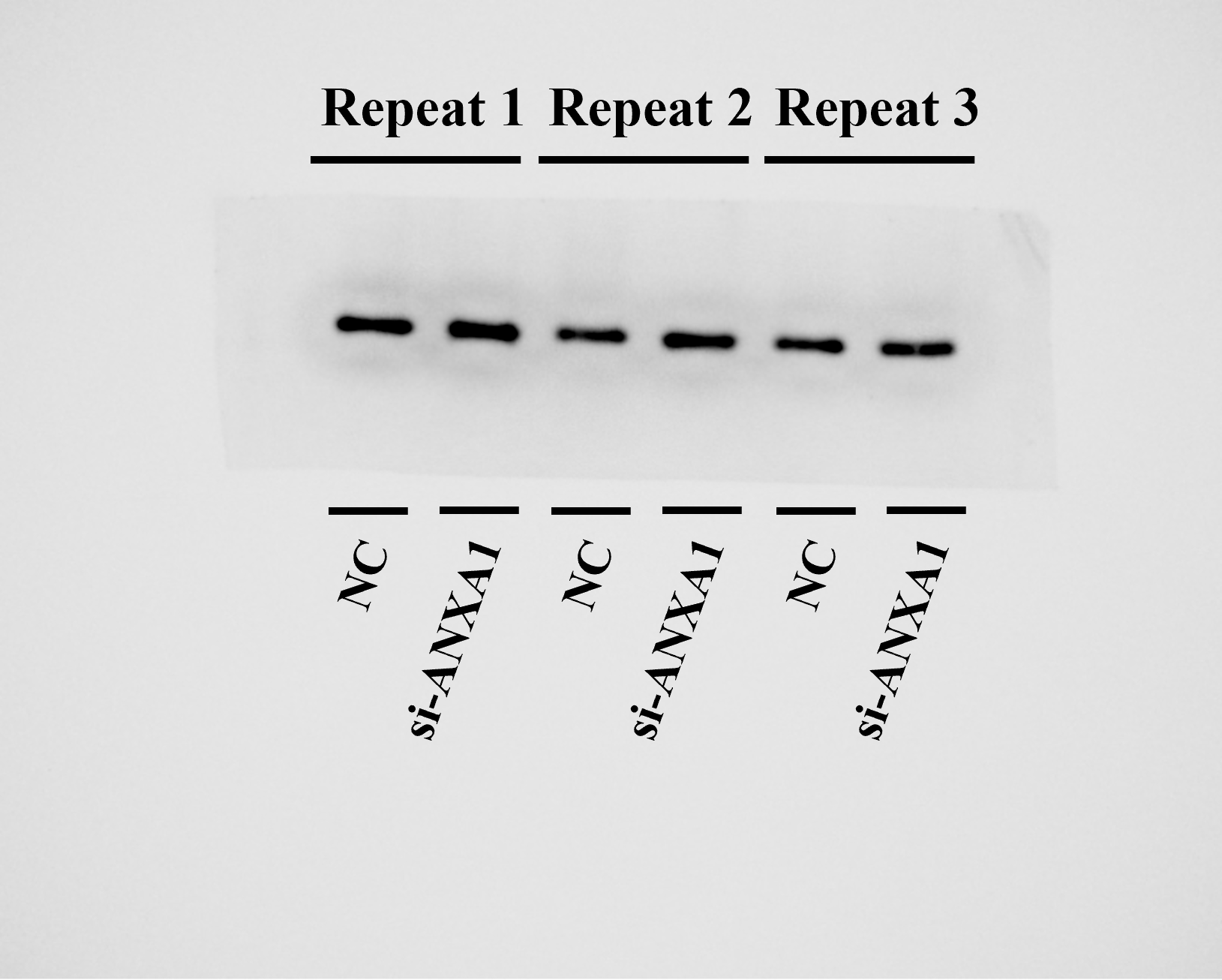

Supplement: Supplementary file 2 [file DataSheet1.zip › Western Blot_raw_images/Figure 3/D/CD9.tif]

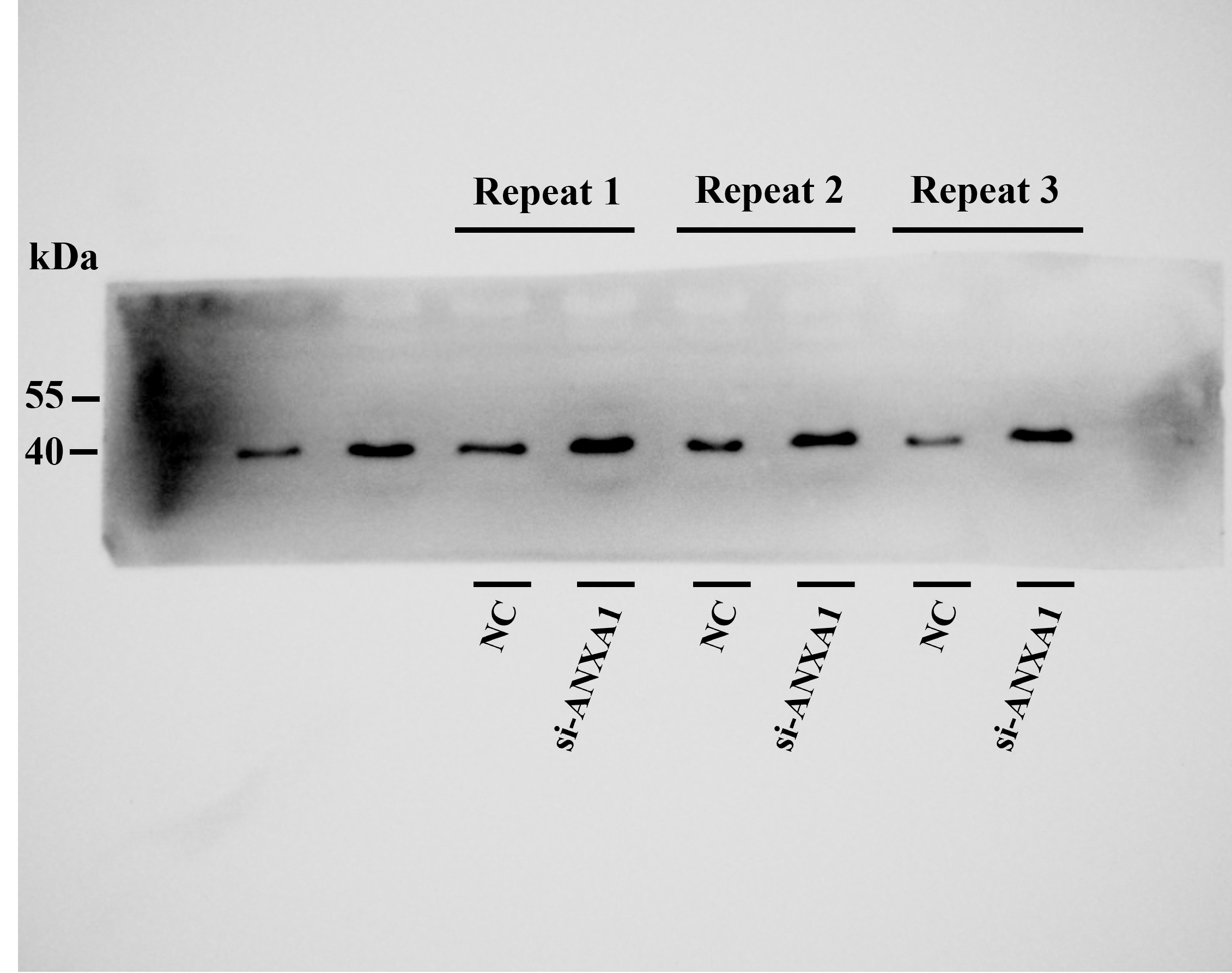

Supplement: Supplementary file 2 [file DataSheet1.zip › Western Blot_raw_images/Figure 3/J/ACP5.tif]

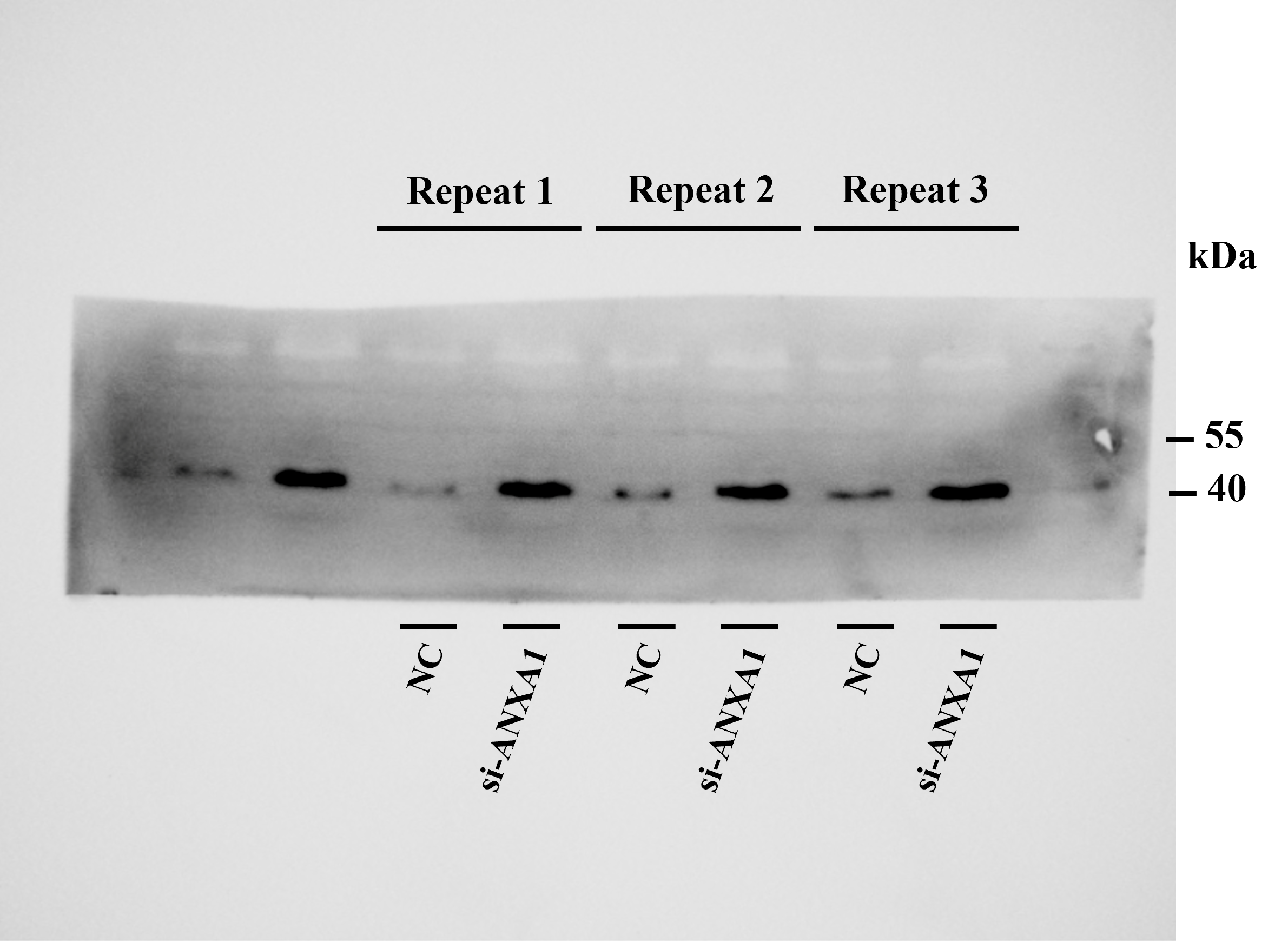

Supplement: Supplementary file 2 [file DataSheet1.zip › Western Blot_raw_images/Figure 3/J/CFOS.tif]

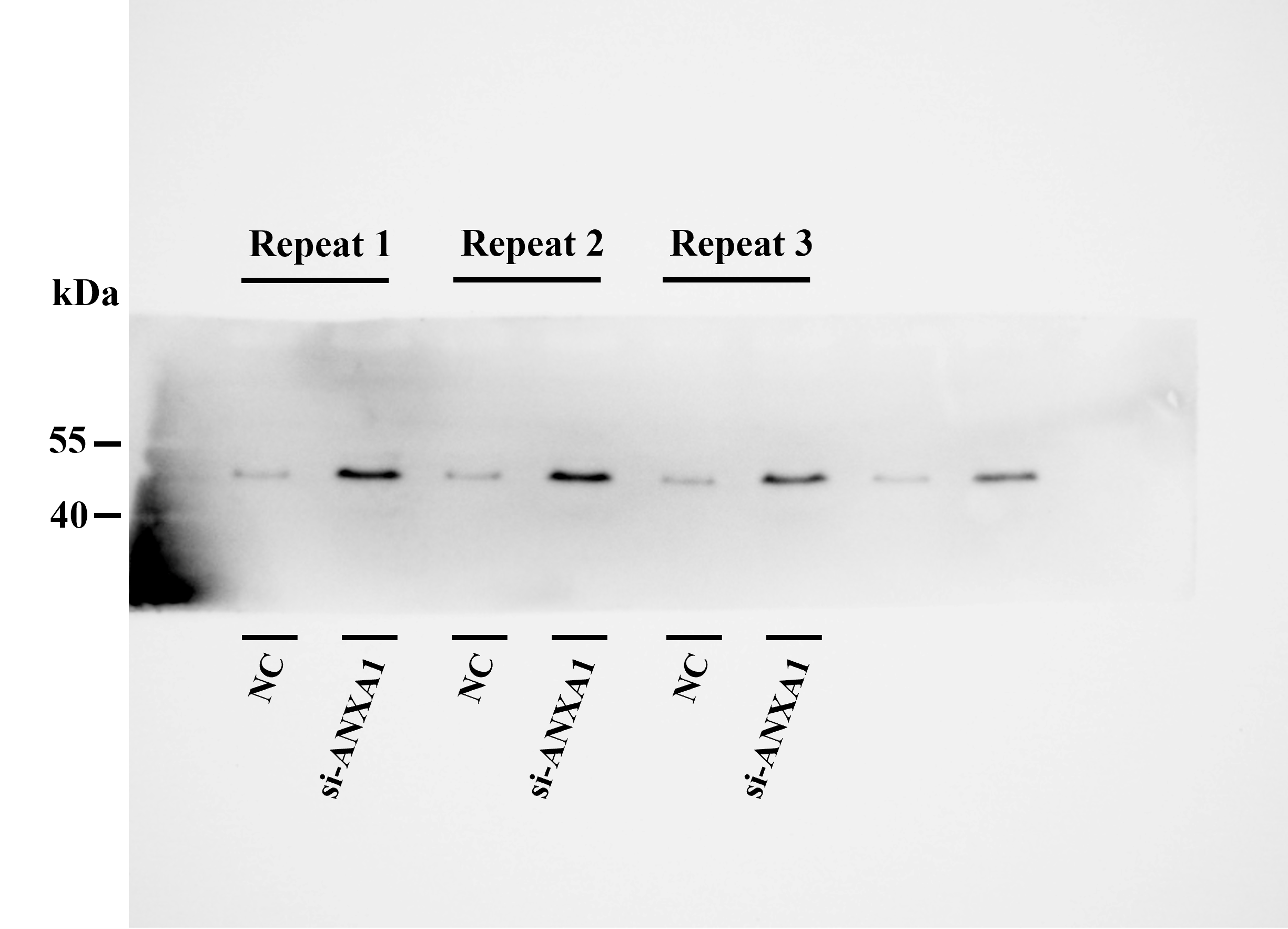

Supplement: Supplementary file 2 [file DataSheet1.zip › Western Blot_raw_images/Figure 3/J/CTSK.tif]

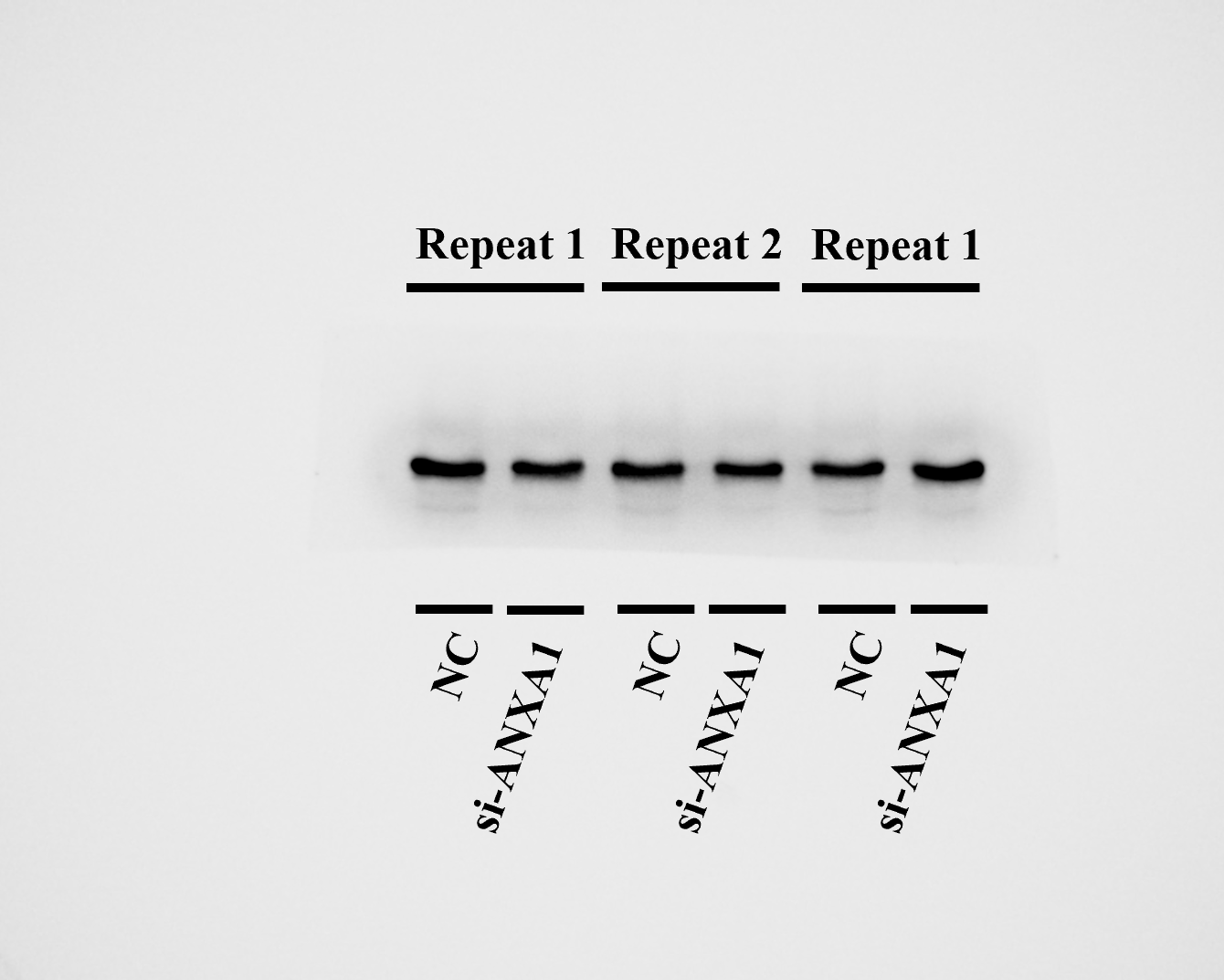

Supplement: Supplementary file 2 [file DataSheet1.zip › Western Blot_raw_images/Figure 3/J/GAPDH.tif]

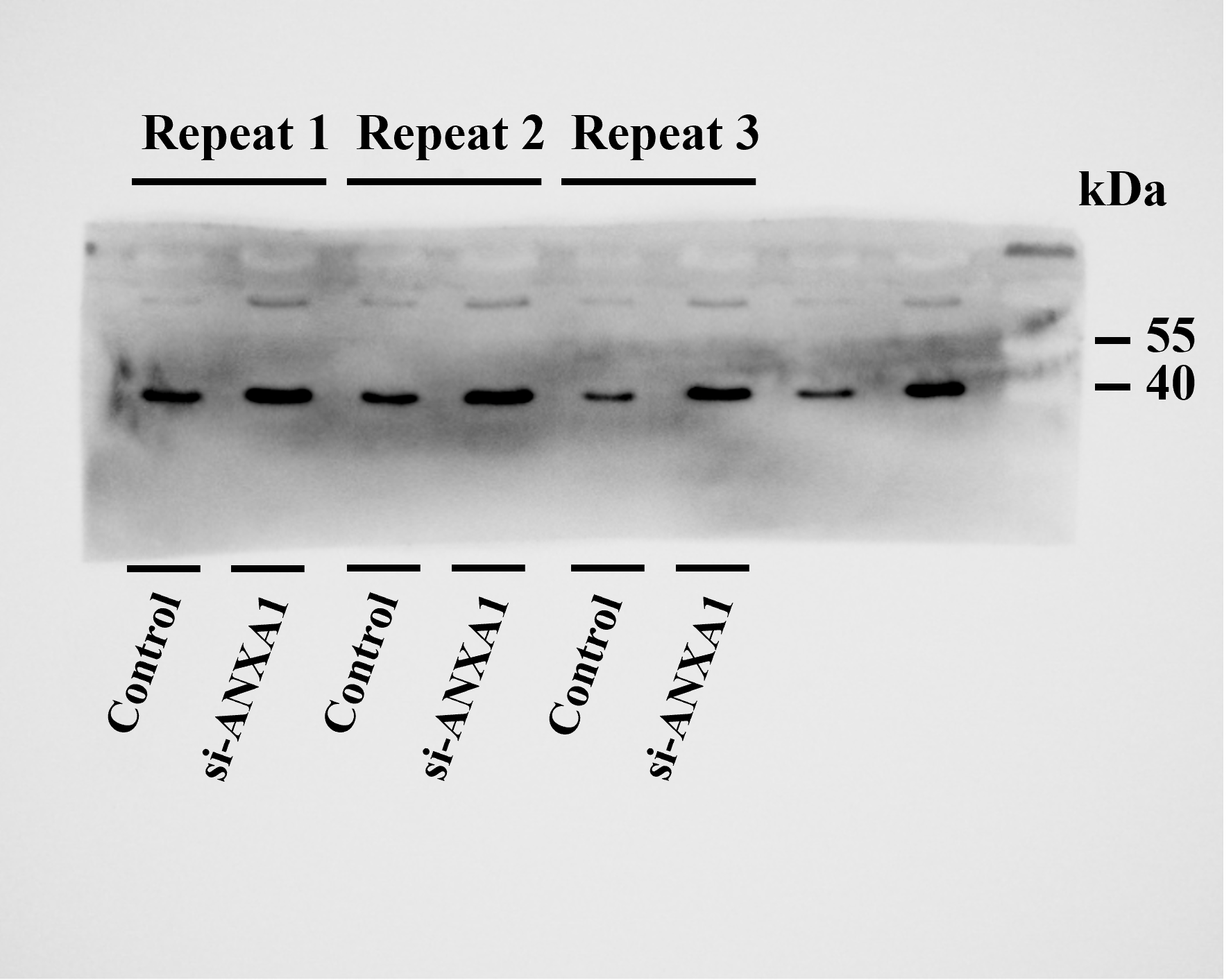

Supplement: Supplementary file 2 [file DataSheet1.zip › Western Blot_raw_images/Figure 4/C/CEBPα.tif]

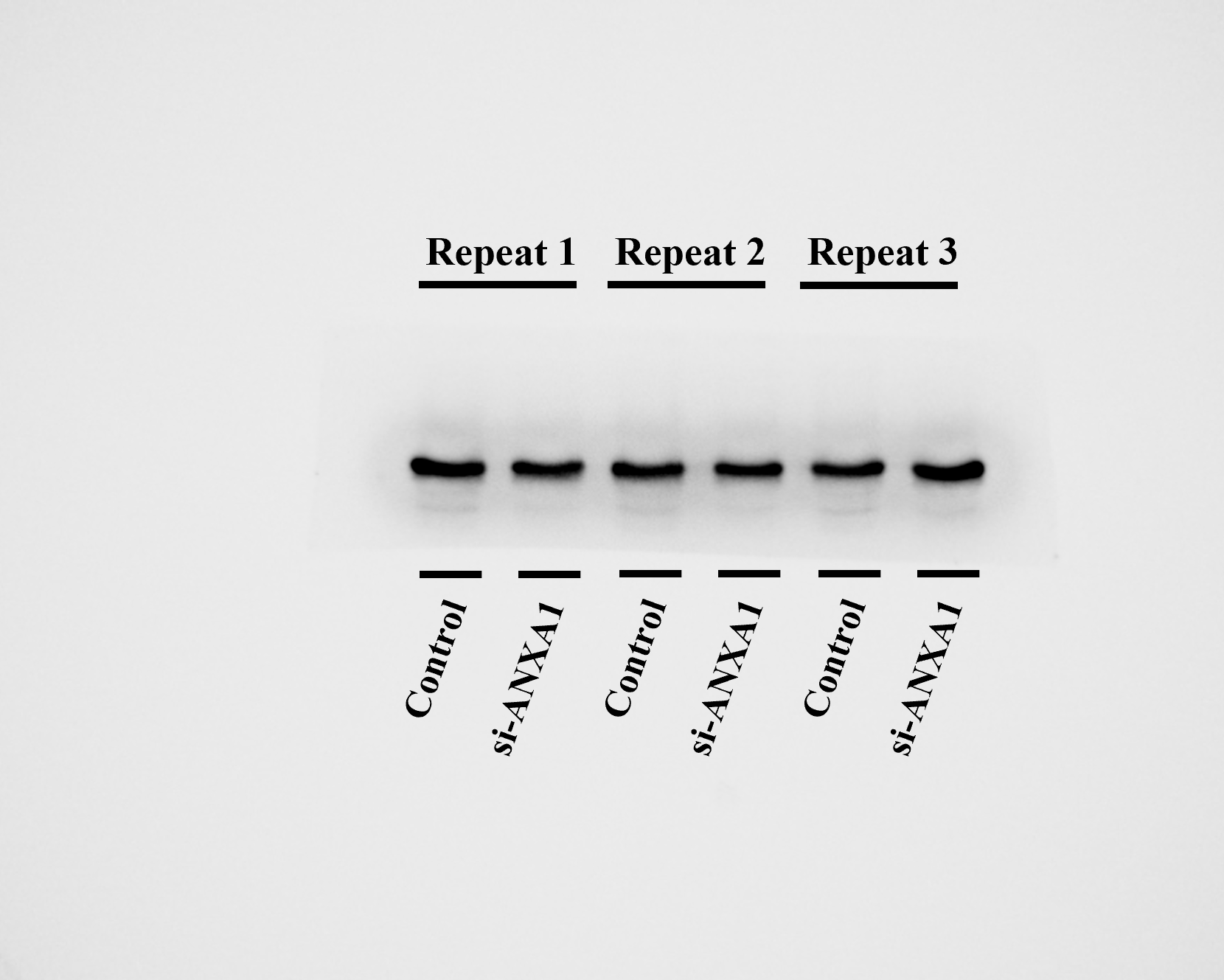

Supplement: Supplementary file 2 [file DataSheet1.zip › Western Blot_raw_images/Figure 4/C/GAPDH.tif]

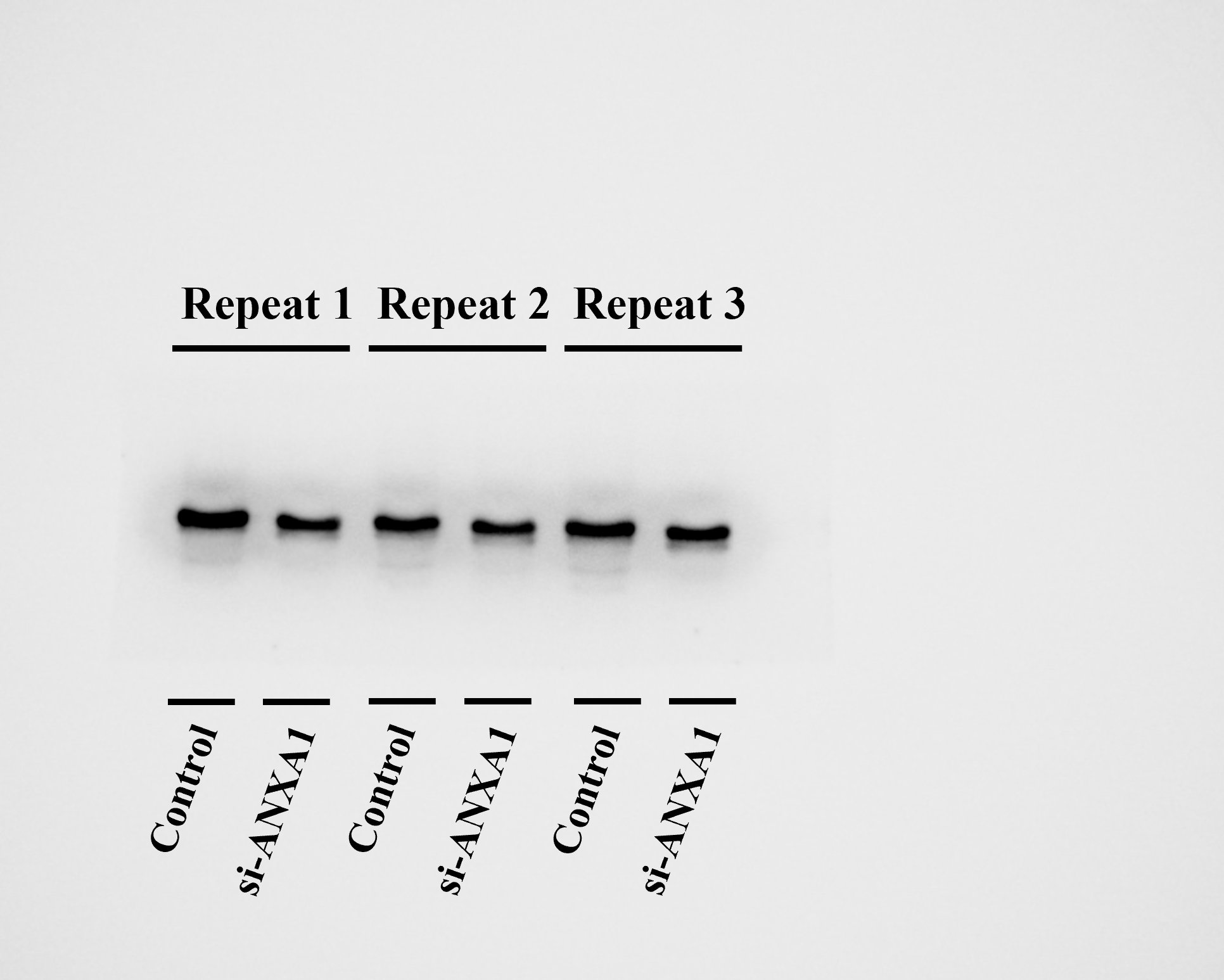

Supplement: Supplementary file 2 [file DataSheet1.zip › Western Blot_raw_images/Figure 4/C/PPARγ.tif]

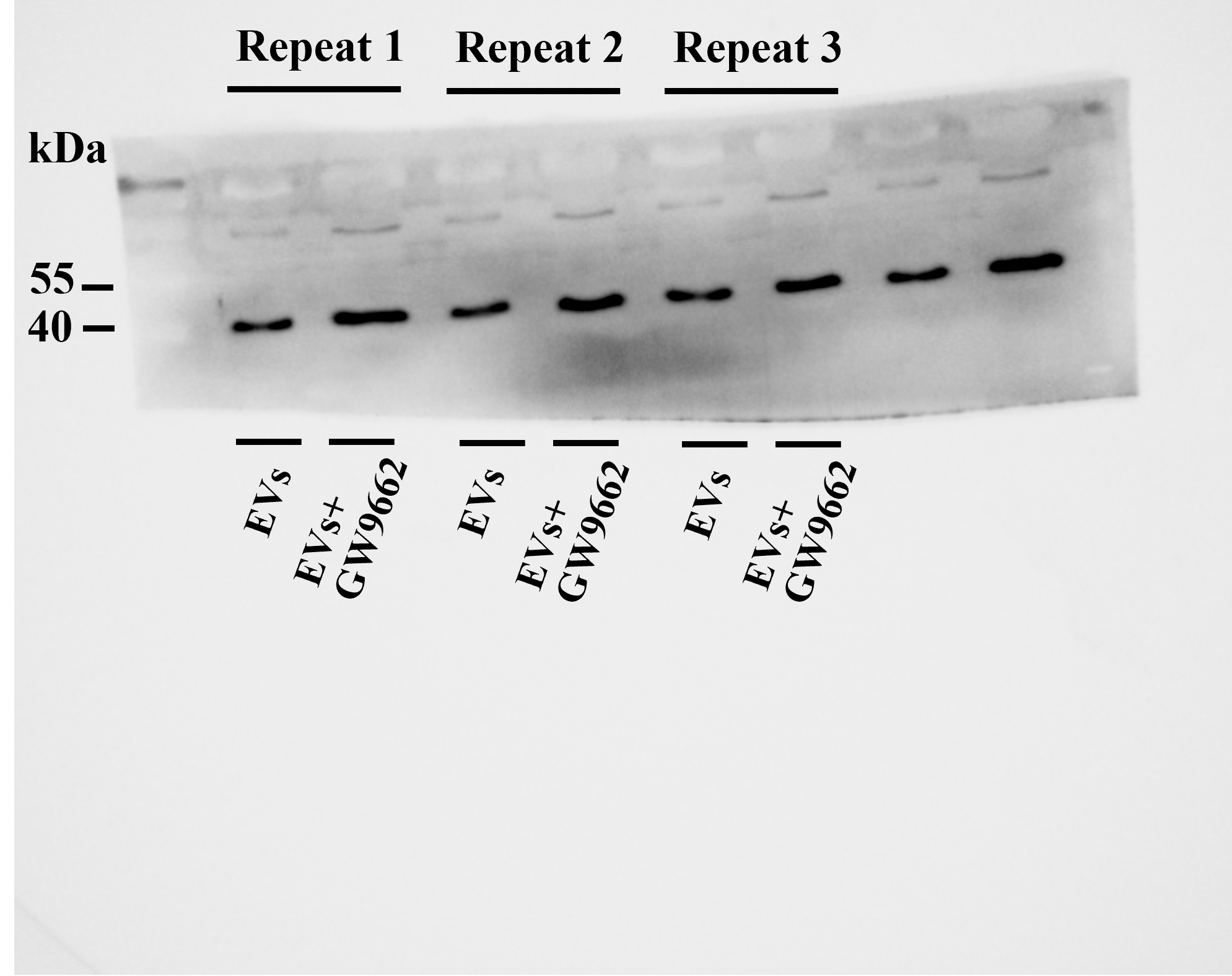

Supplement: Supplementary file 2 [file DataSheet1.zip › Western Blot_raw_images/Figure 4/K/CEBPα.tif]

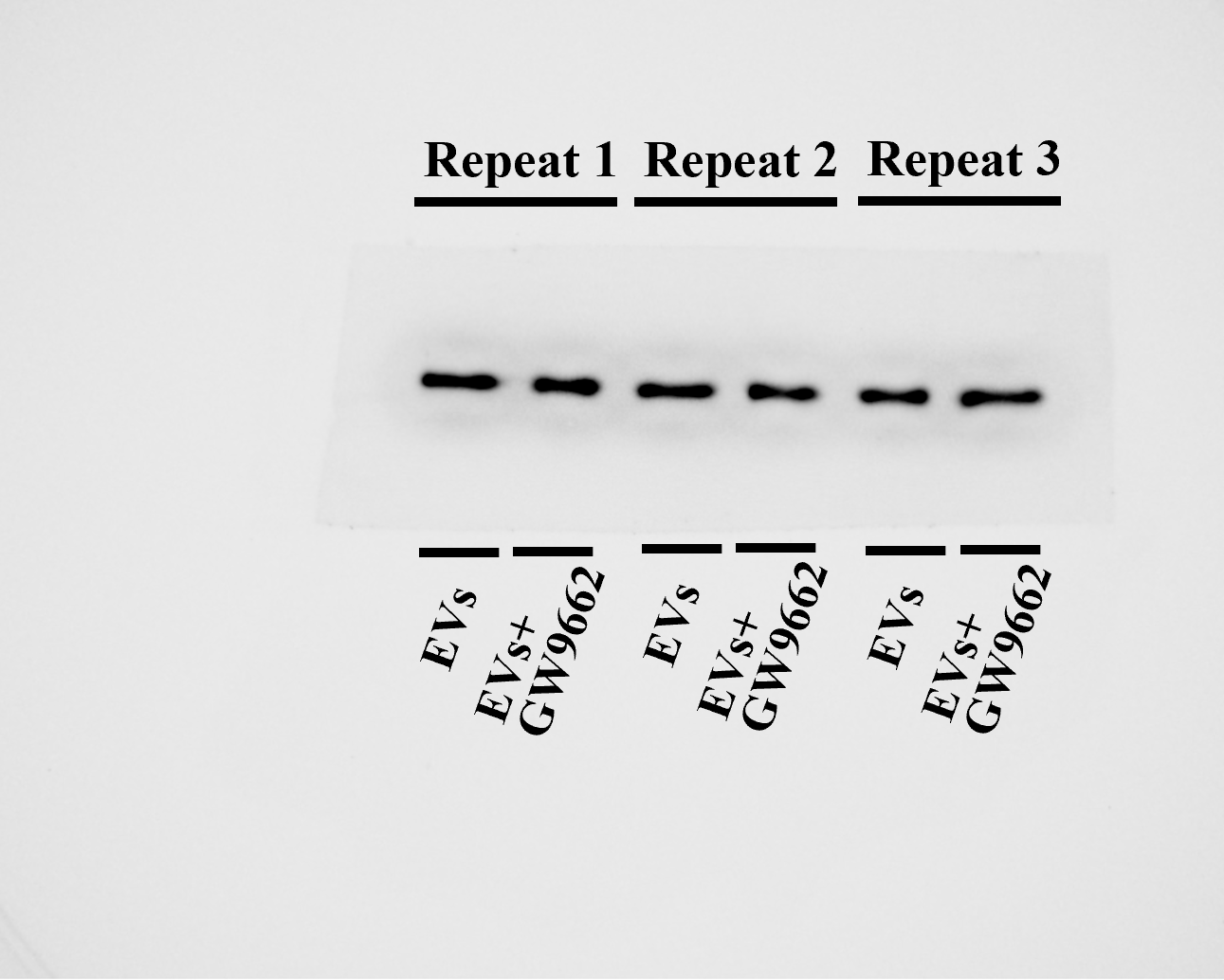

Supplement: Supplementary file 2 [file DataSheet1.zip › Western Blot_raw_images/Figure 4/K/GAPDH.tif]

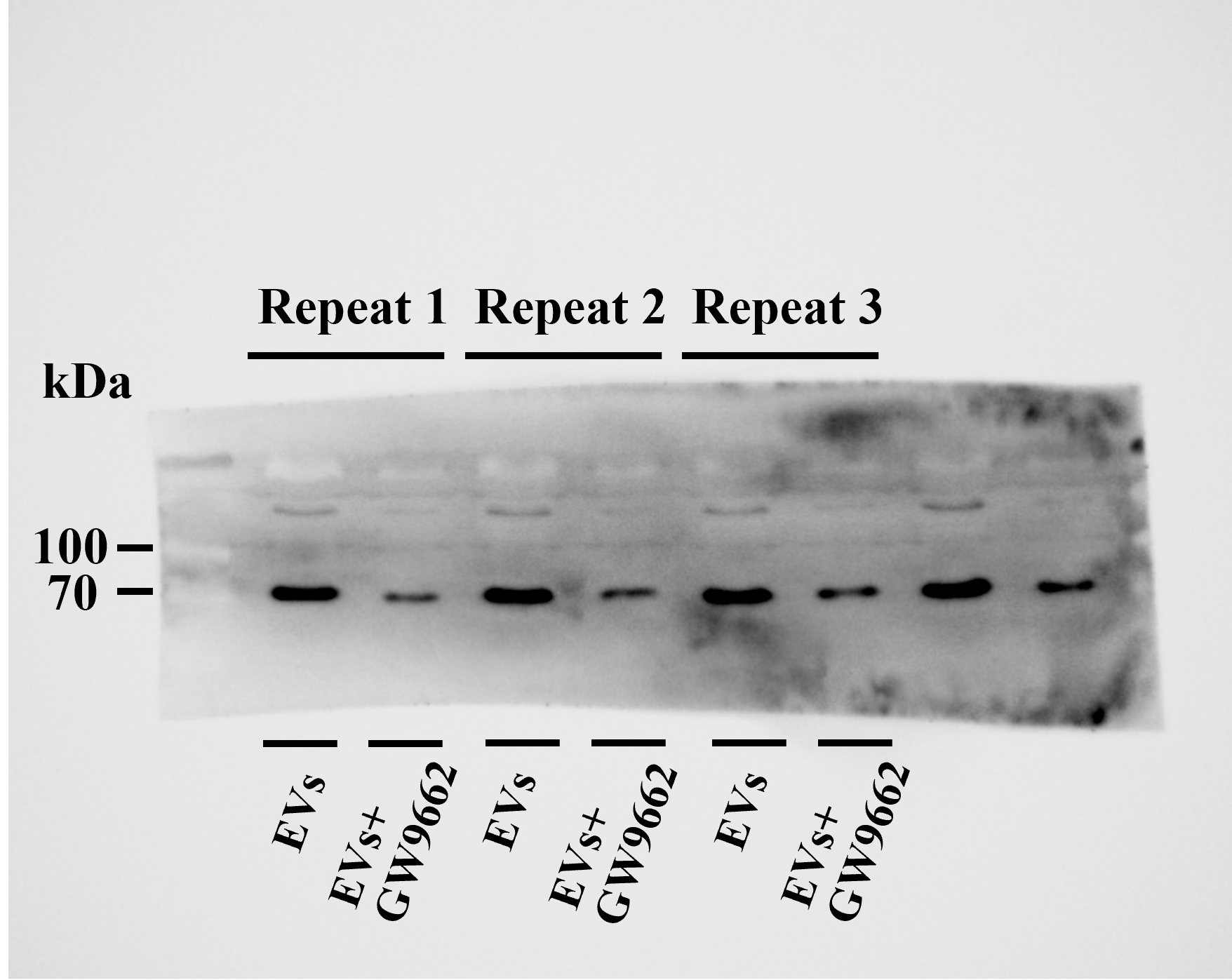

Supplement: Supplementary file 2 [file DataSheet1.zip › Western Blot_raw_images/Figure 4/K/PPARγ.tif]

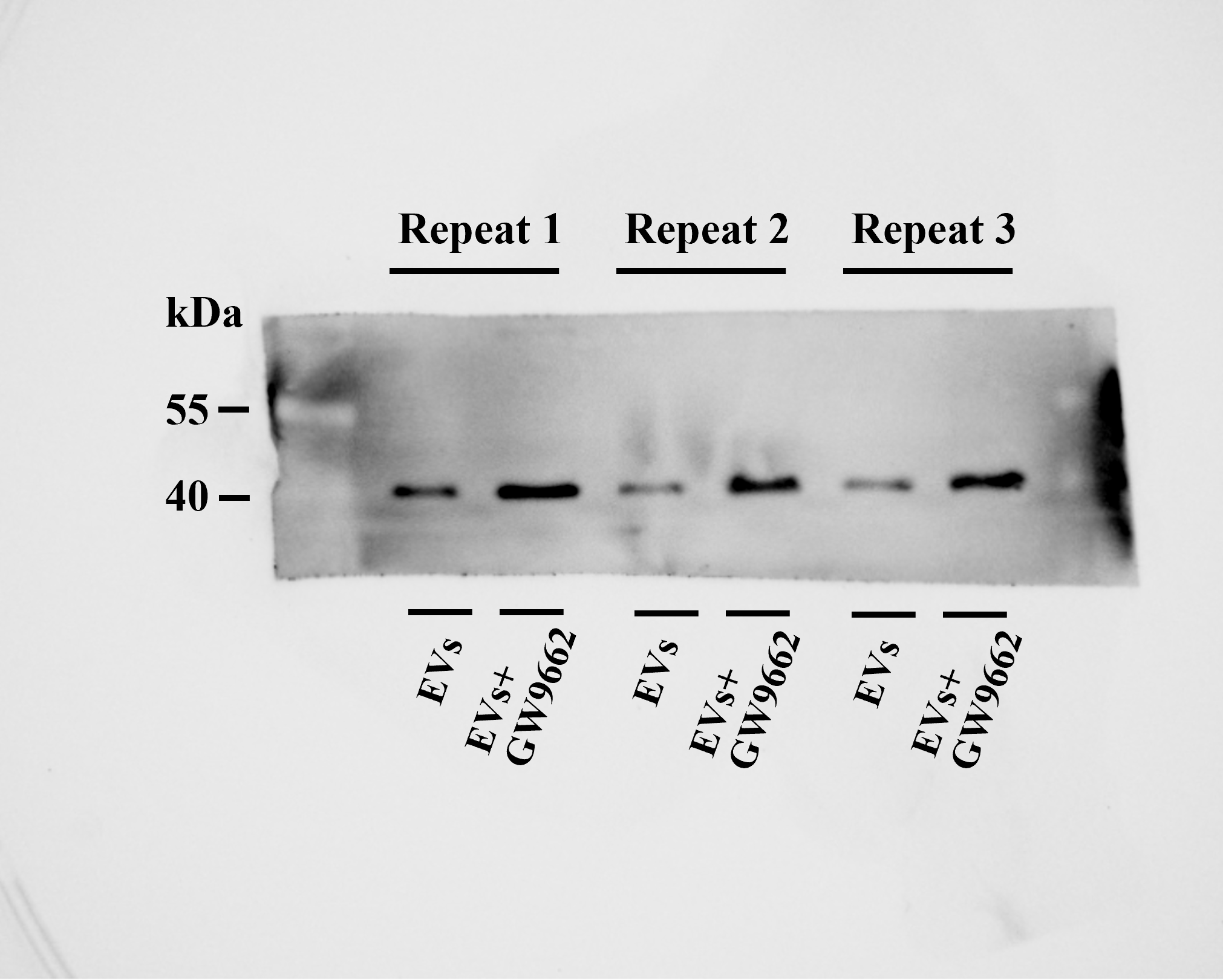

Supplement: Supplementary file 2 [file DataSheet1.zip › Western Blot_raw_images/Figure 4/O/ACP5.tif]

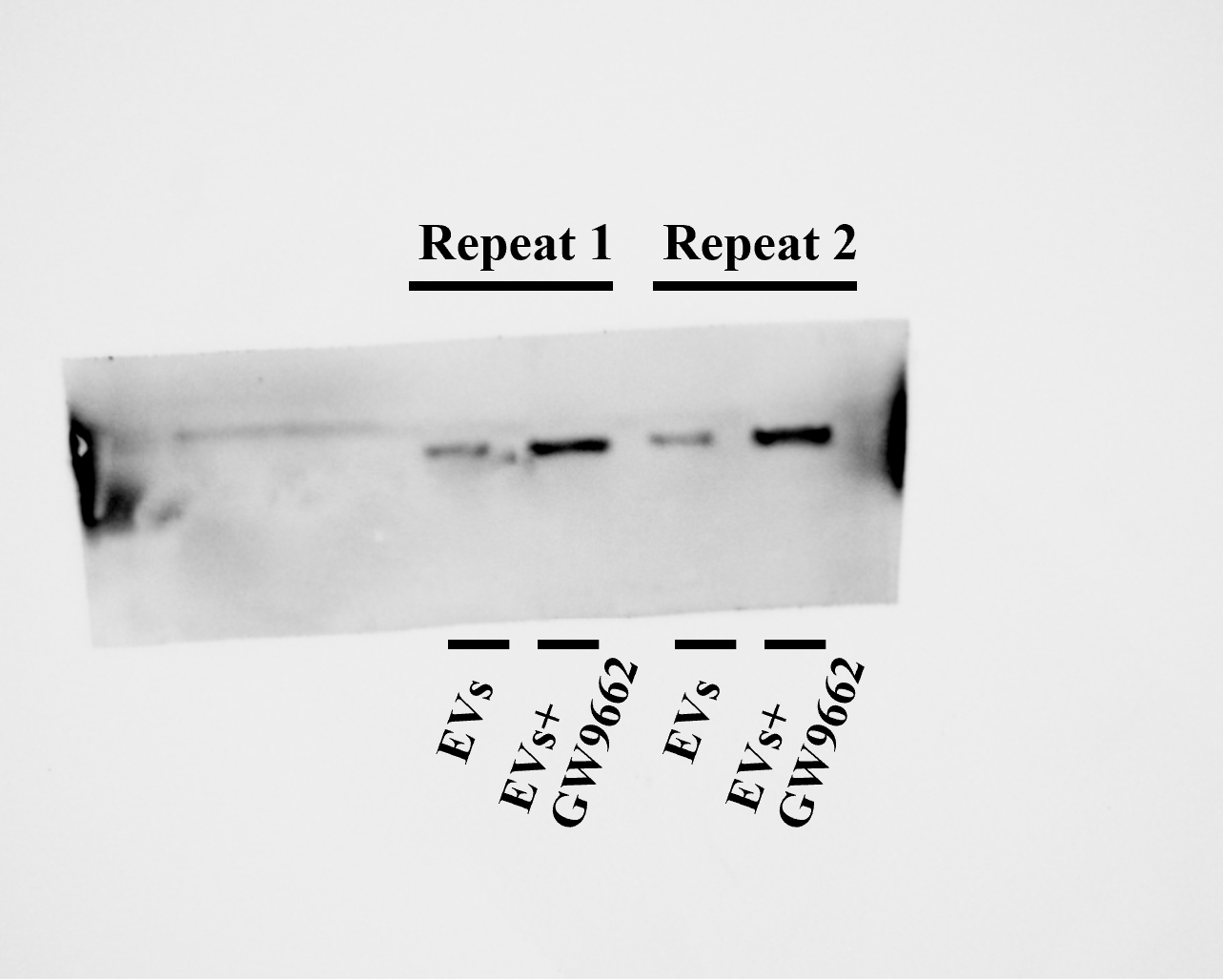

Supplement: Supplementary file 2 [file DataSheet1.zip › Western Blot_raw_images/Figure 4/O/CFOS (1).tif]

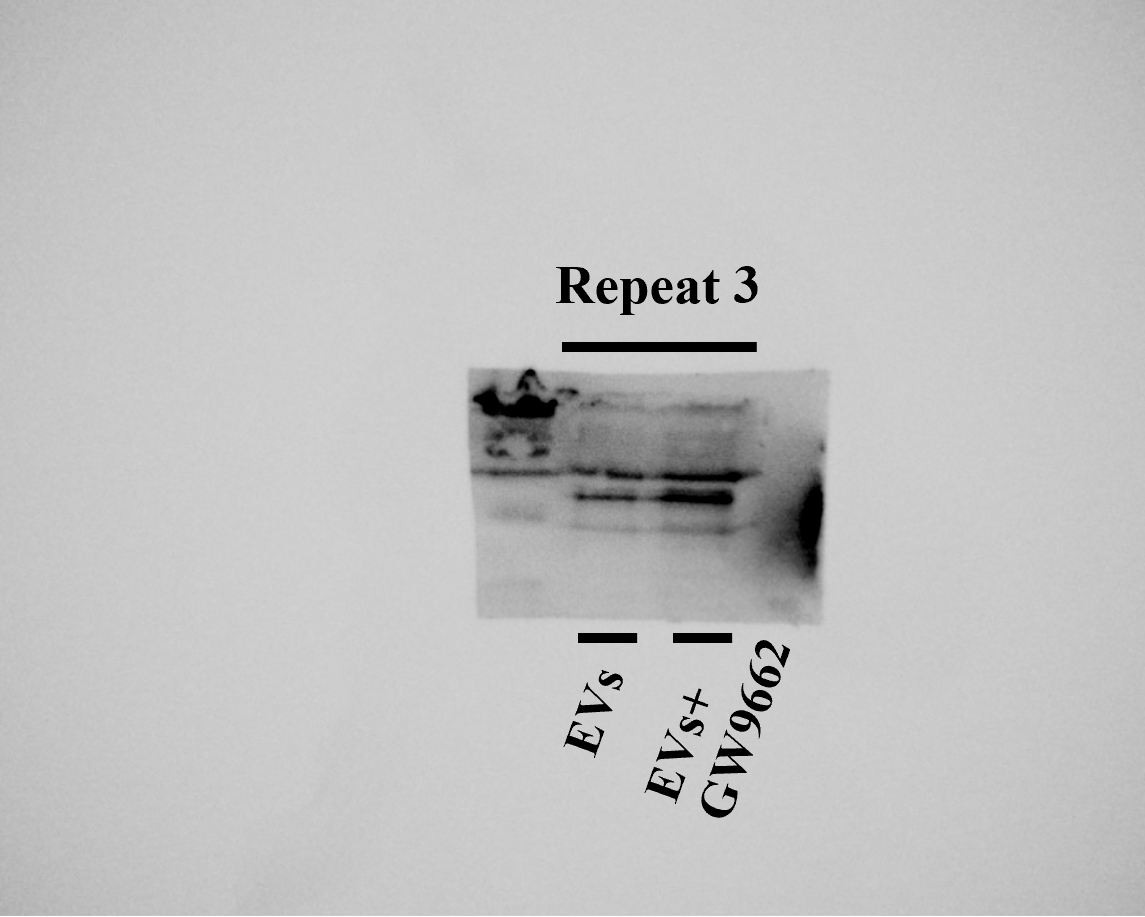

Supplement: Supplementary file 2 [file DataSheet1.zip › Western Blot_raw_images/Figure 4/O/CFOS (2).tif]

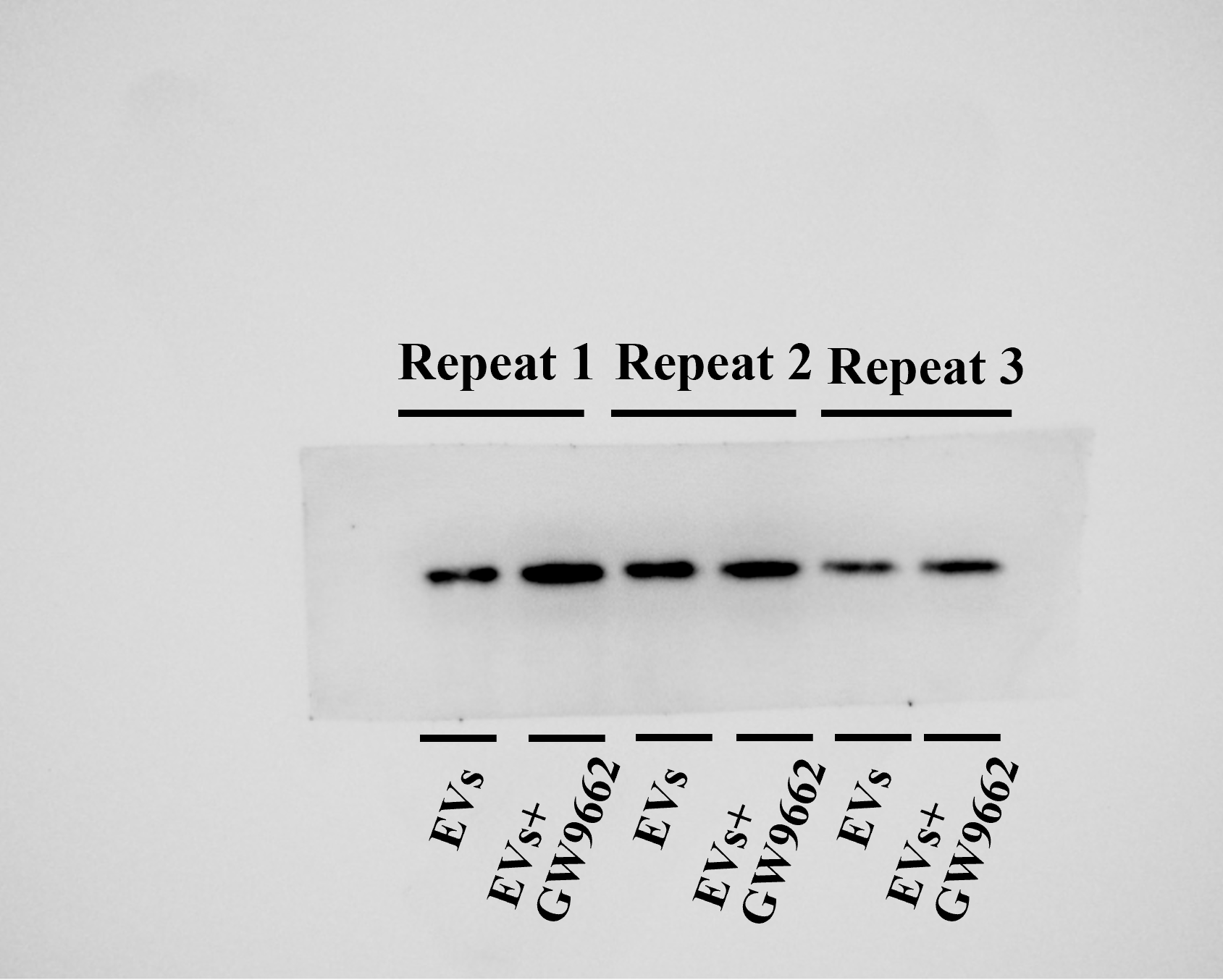

Supplement: Supplementary file 2 [file DataSheet1.zip › Western Blot_raw_images/Figure 4/O/CTSK.tif]

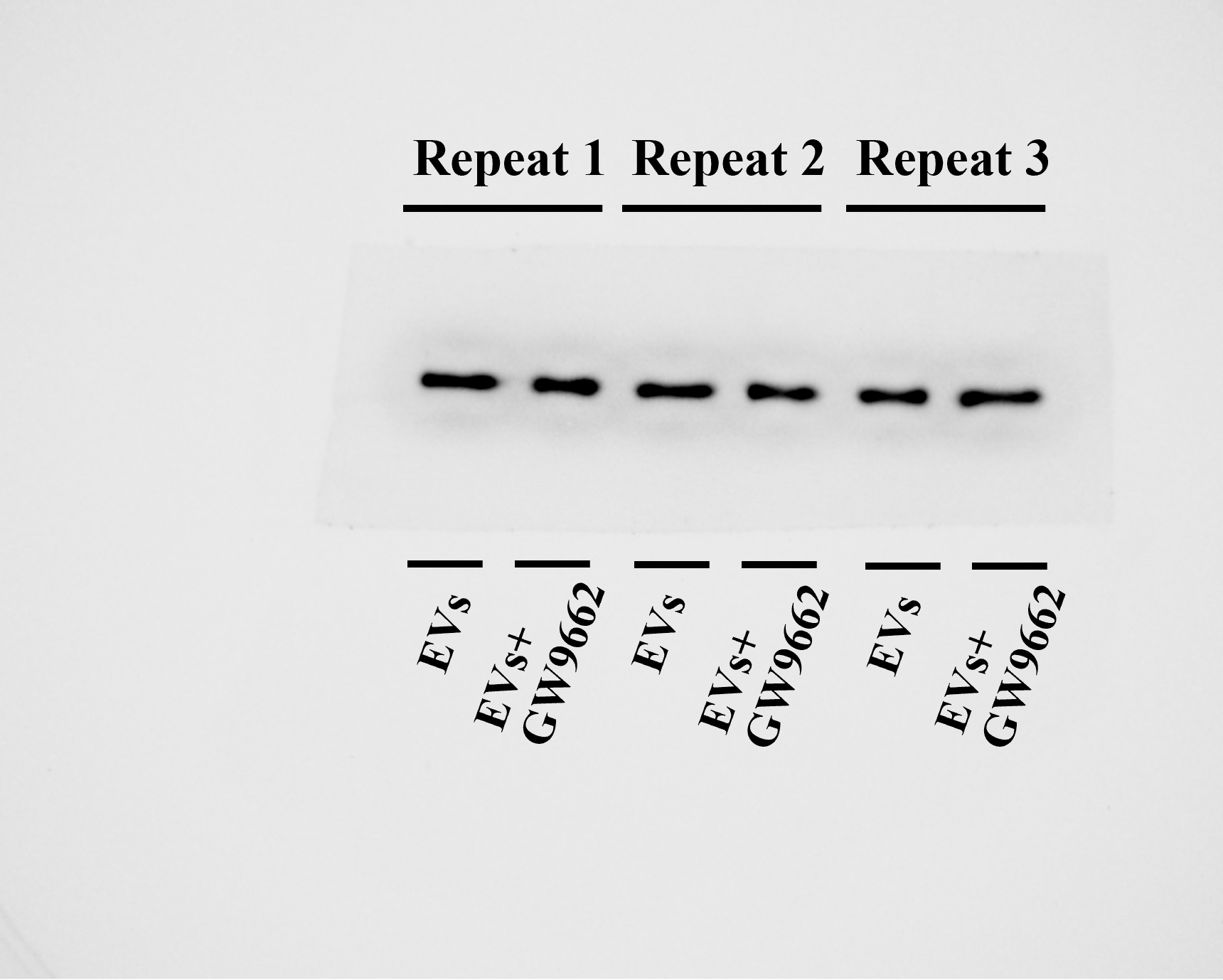

Supplement: Supplementary file 2 [file DataSheet1.zip › Western Blot_raw_images/Figure 4/O/GAPDH.tif]

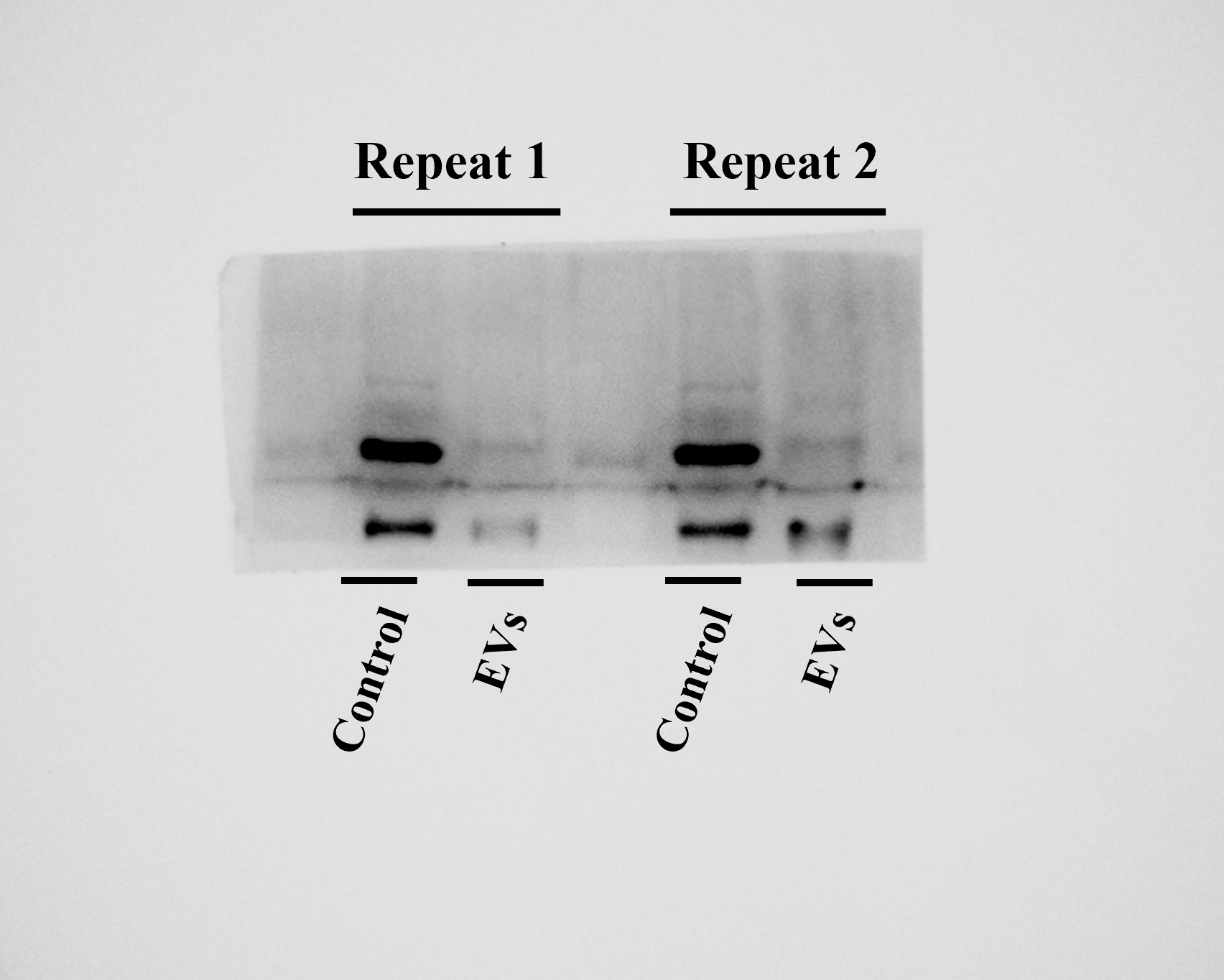

Supplement: Supplementary file 2 [file DataSheet1.zip › Western Blot_raw_images/Figure S2/D/Calnexin.tif]

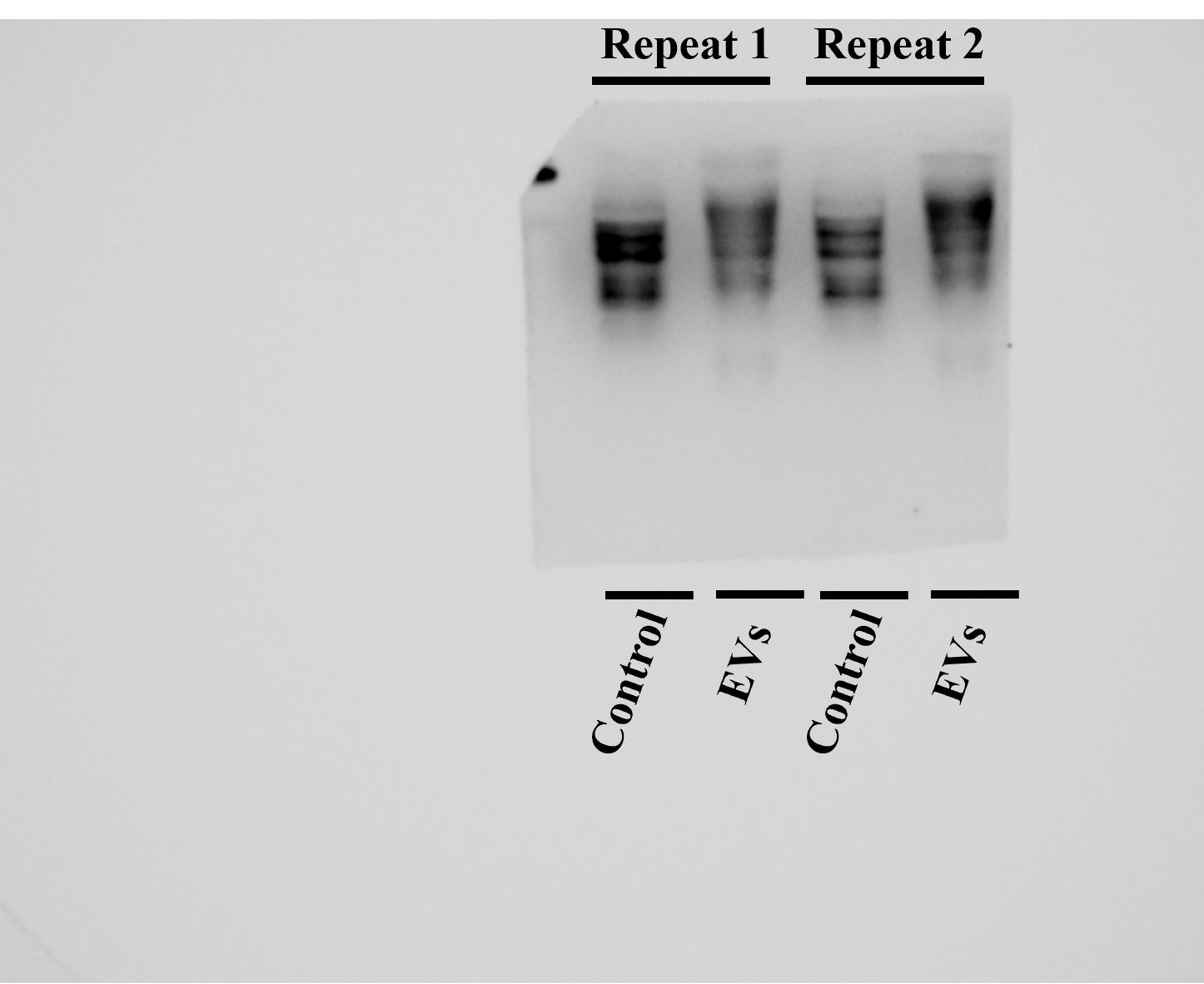

Supplement: Supplementary file 2 [file DataSheet1.zip › Western Blot_raw_images/Figure S2/D/CD63.tif]

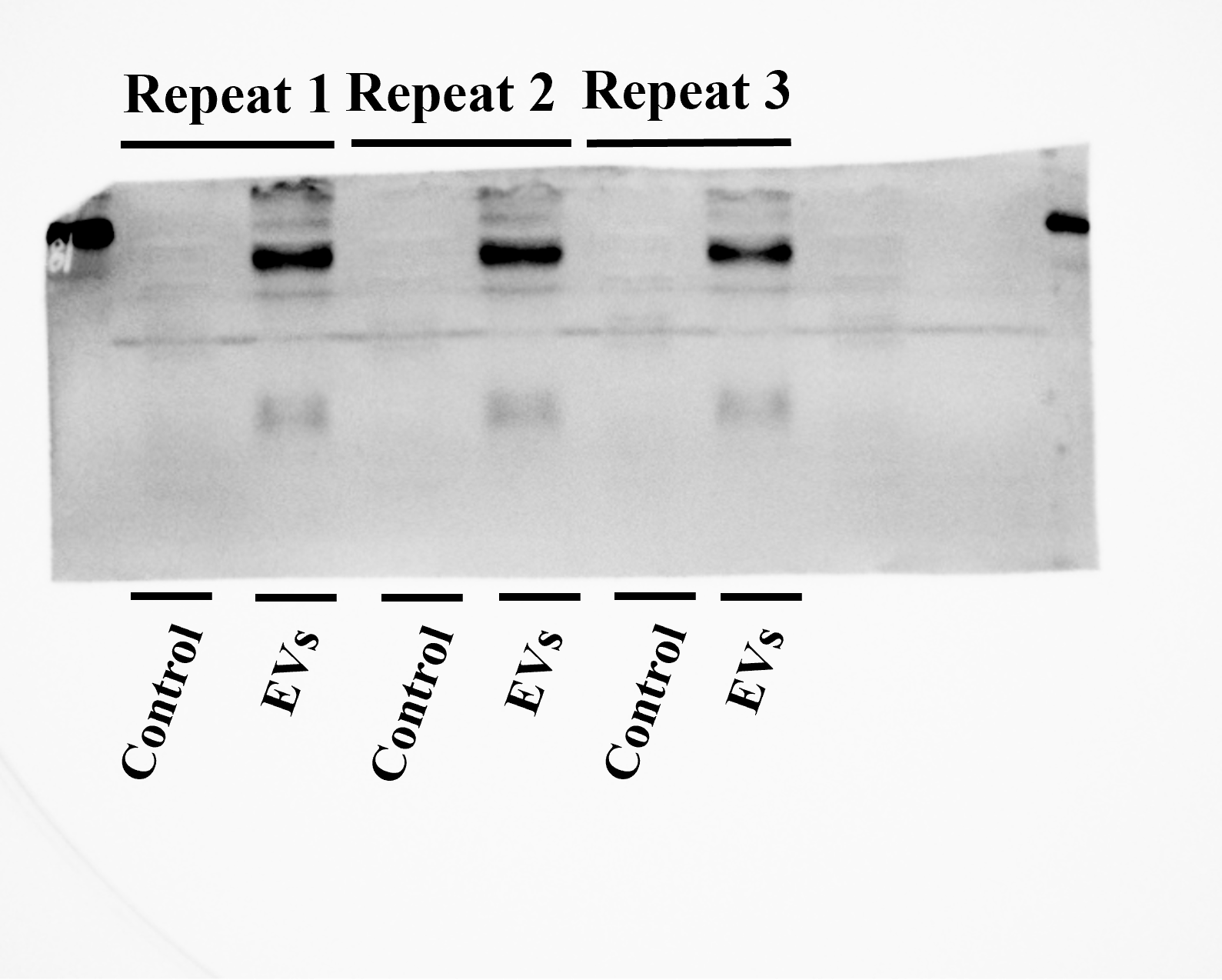

Supplement: Supplementary file 2 [file DataSheet1.zip › Western Blot_raw_images/Figure S2/D/CD81.tif]

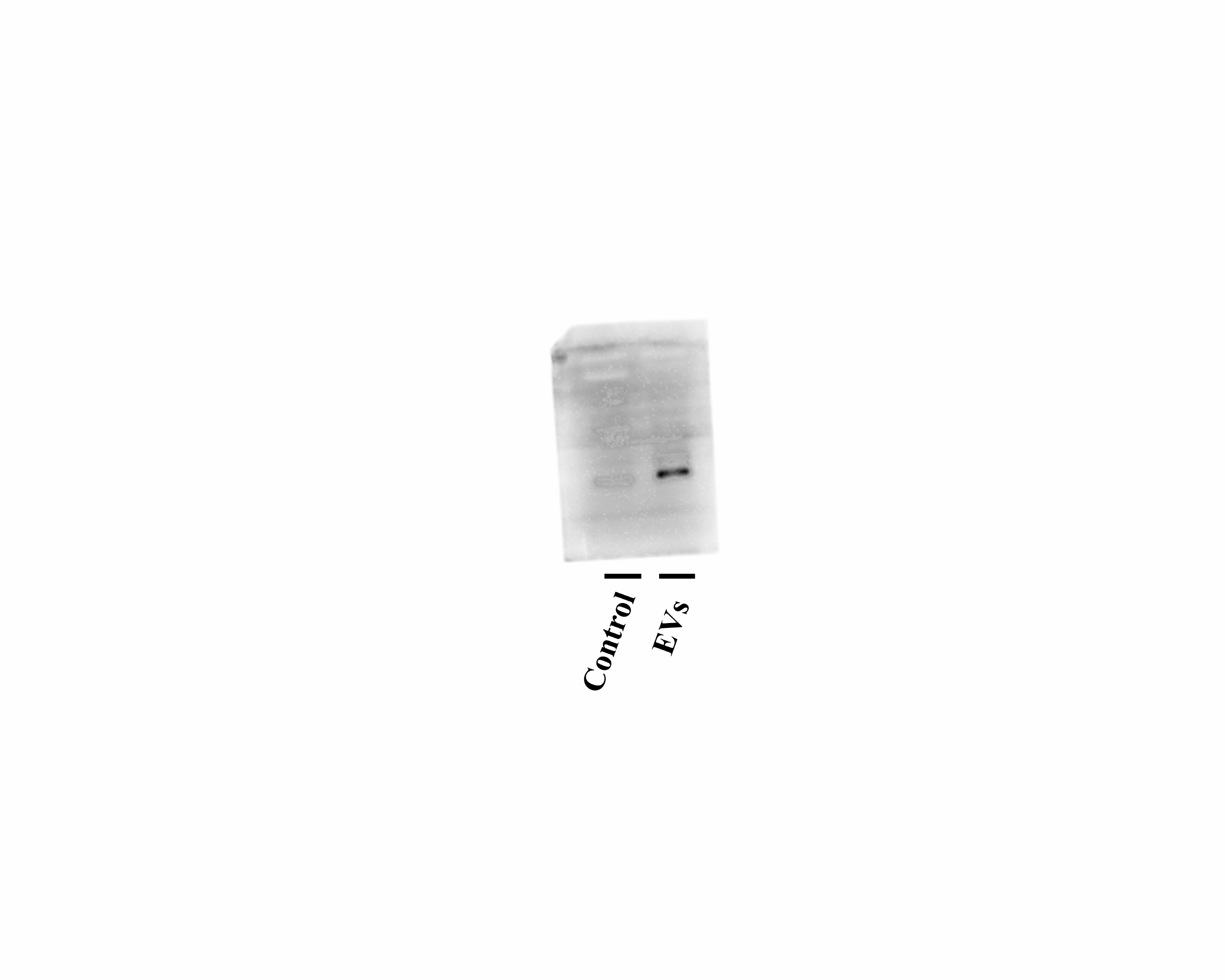

Supplement: Supplementary file 2 [file DataSheet1.zip › Western Blot_raw_images/Figure S2/D/CD9.tif]
